# New Prospects in the Inhibition of Monoamine Oxidase‑B (MAO-B) Utilizing Propargylamine Derivatives for the Treatment of Alzheimer’s Disease: A Review

**DOI:** 10.1021/acsomega.5c00134

**Published:** 2025-06-16

**Authors:** Filippos Panteleimon Chatzipieris, Athanasios Kokkalis, Nikitas Georgiou, Errikos Petsas, Ektoras Vasileios Apostolou, Georgios C. Vougioukalakis, Thomas Mavromoustakos

**Affiliations:** Laboratory of Organic Chemistry, Department of Chemistry, 68993 National and Kapodistrian University of Athens, 15771 Athens, Greece

## Abstract

It is well-known that monoamine oxidase (MAO) plays a pivotal role in neurodegeneration and the inhibition of this enzyme can manifest antidepressant properties as well as have a positive impact in Alzheimer’s and Parkinson’s diseases. MAO has two isoforms: MAO-A and MAO-B. The main hMAO-B inhibitors used for the treatment of Alzheimer’s and Parkinson’s diseases, encompass a terminal triple bond in their structure, which provides their potency. Recently, a new class of inhibitors has emerged, bearing the carbon–carbon triple bond not necessarily at the end of the chain. In this review, the structure and physiological function of the MAO enzymes is discussed, the general synthetic procedures of propargylamines, as well as their mechanism of inhibition. Moreover, it is highlighted the current development and discovery of potential hMAO-B inhibitors from propargylamine scaffolds, showcasing their structure–activity relationships (SARs) with the enzyme. Conformational relationships’ analysis is performed as well. Induced fit docking is performed, also, to the most potent compounds revealed in order to assess their binding energy and interactions with the enzyme. Finally, molecules which do not contain a propargylamine moiety in their structure were studied and compared against a known hMAO-B inhibitor, deprenyl. From the superimposition results of these molecules with deprenyl, as well as the interactions of the molecules with the amino acids of the active site of hMAO-B, it appears that these compounds have several similarities with deprenyl, opening new paths for the creation of novel molecules against Alzheimer’s disease (AD).

## Graphical abstract

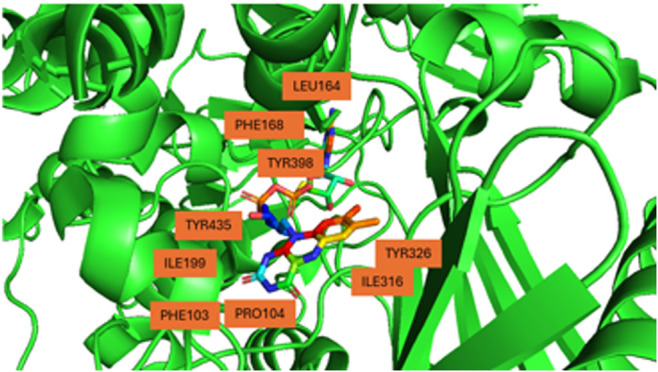

## 1 Introduction

Alzheimer’s disease (AD), is a well-known progressive form of neuronal cell degeneration, which influences older humans and is estimated to affect 139 million people by 2050.^1^ AD is the leading cause of dementia, marked by significant cognitive decline, which includes impairments in memory, language, intellectual abilities, and visual-spatial skills.^2^ As the disease progresses, neuropsychiatric symptoms become more pronounced, while daily functioning declines.^3^ In 2021, official Alzheimer’s disease death certificates documented 119,399 deaths. During 2020 and 2021, when COVID-19 became one of the top ten causes of death, Alzheimer’s ranked as the seventh leading cause of death in the United States. From 2000 to 2021, deaths from stroke, heart disease, and HIV declined, while reported deaths from Alzheimer’s disease rose by over 140%. Moreover, in 2023, over 11 million family members and other unpaid caregivers provided an estimated 18.4 billion hours of care to individuals with Alzheimer’s or other dementias, facing a higher risk of emotional distress and negative mental and physical health outcomes.^4^

Pathologically, AD is characterized by the accumulation of intracellular neurofibrillary tangles (NFTs) and extracellular senile plaques containing amyloid-β (Aβ) proteins. These, along with neuronal death and brain atrophy, are the defining features of the condition. In AD, the brain also exhibits an “inflammatory” cascade, even in the early stages. This cascade triggers the activation of microglia and astroglia, which in turn activate various signaling pathways^5,6^ that produce inflammatory responses, such as reactive oxygen species (ROS) and cytokines formation, leading to oxidative stress.^7^

Monoamine oxidase (MAO) is an enzyme attached to the mitochondria, with high expression levels in both neuronal and gastrointestinal tissues. It exists in two isoforms: MAO-A and MAO-B. These isoforms share considerable sequence similarity, but differ in their substrate and inhibitor recognition sites, as well as in their tissue distribution. They catalyze the oxidative deamination of various monoamines and play key roles in metabolizing released neurotransmitters. Changes in neurotransmitter levels in the brain are linked to the biochemical pathology of several neurological disorders, including depression, Alzheimer’s disease, and Parkinson’s disease.^8–10^ Mitochondrial-bound monoamine oxidases are primarily found in the human brain. However, MAO-A is also present in the placenta, heart, and intestines, while MAO-B is found in brain glial cells, platelets, and liver cells. Additionally, MAOs play a role in regulating mood, motor function, brain and motivational processes.^11–13^

In AD brains, the expression of MAO-B is increased in the hippocampus and cerebral cortex compared to healthy brains.^14,15^ A significantly higher level of active MAO-B is found in reactive astrocytes surrounding amyloid-β deposits of the hippocampus and frontal cortex of the brains with AD.^16^ The overexpression of MAO-B in astrocytes is thought to promote the excessive metabolism of monoamines, leading to an increased production of free radicals and hydrogen peroxide (H_2_O_2_). This, in turn, may contribute to the neurodegenerative processes associated with AD. This mechanism appears to be an early, ongoing event in AD that persists throughout the progression of the disease.^17^

Moreover, MAO-B activation plays a role in the formation of amyloid-β plaques. Research on the pathophysiology of Alzheimer’s disease (AD) has indicated that oxidative damage is a key feature of the condition. In AD patients, oxidative stress promotes the formation of amyloid-β plaques. Excessive monoamine oxidase activity promotes both amyloidogenic and nonamyloidogenic cleavage of amyloid precursor protein (APP) by directly stimulating the activity of β-secretase and γ-secretase, ultimately resulting in abnormal amyloid plaque formation (Figure 1).^18,19^

**Figure 1:**
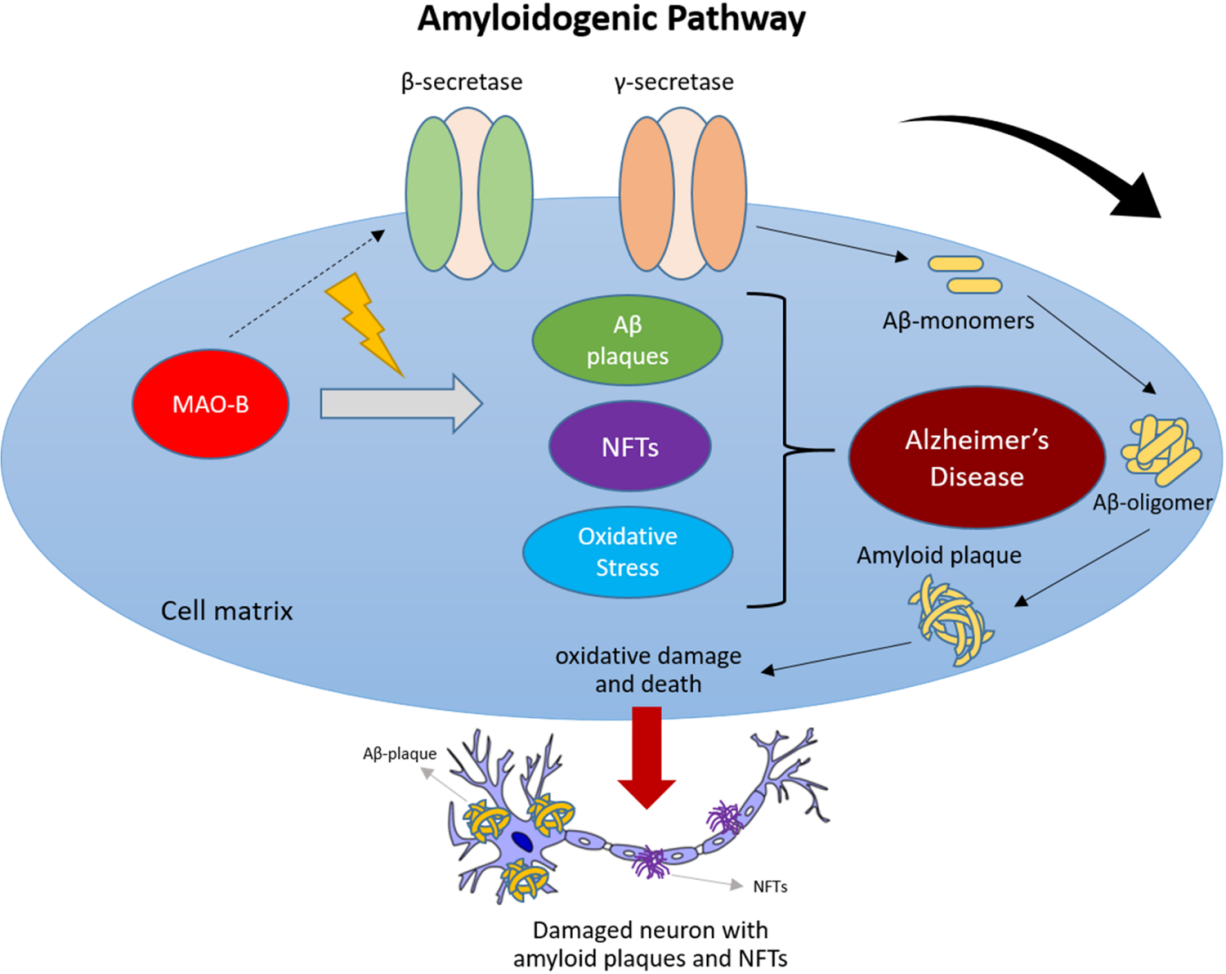
Activation of monoamine oxidase B (MAO-B) promotes both amyloidogenic and nonamyloidogenic cleavage of amyloid precursor protein (APP) by directly stimulating the activity of β-secretase and γ-secretase, ultimately resulting in abnormal amyloid plaque formation.

Given the assumed role of MAO-B in AD, inhibiting its expression could be expected to reduce oxidative stress and neurodegeneration, potentially slowing the progression of the disease. Propargylamines have garnered considerable interest in recent decades because of their distinctive structural features, which, among other things, enable further functionalization. The propargylamine core is present in compounds of significant pharmaceutical value, like pargyline and selegiline, which are used to treat neurodegenerative diseases (NDs) such as depression, Parkinson’s disease, and potentially Alzheimer’s disease.^20–23^ In this review, we will examine the newly developed propargylamine derivatives from 2020 to 2024, which can be used for the treatment of the symptoms of AD through the inhibition of the hMAO-B enzyme and an interesting new class of them which holds the triple bond in a nonterminal region. The general procedures of propargylamine synthesis are established, alongside the compounds’ structure–activity relationships with MAO-B and conformational relationships’ analysis is performed. We have also outlined that new avenues are possible for finding novel structures against AD. This was achieved by investigating molecules which do not contain a propargylamine moiety in their structure and comparing them with a known hMAO-B inhibitor, deprenyl, via computational chemistry techniques.

### 1.1 Human Monoamine Oxidases (MAO-A and MAO-B)

Human monoamine oxidases (MAOs) are enzymes that contain flavin adenine dinucleotide (FAD) and are found outside the mitochondrial membrane. They play a key role in the oxidative deamination of a range of endogenous and dietary amines.^24^ Two are the main MAO isoforms; MAO-A and MAO-B.^25^ Research has indicated that the oxidative deamination carried out by MAOs leads to elevated levels of aldehydes and hydrogen peroxide. The increase of these neurotoxic byproducts can stimulate the formation of reactive oxygen species, potentially causing neuronal damage and cell death.^26,27^ In the neuronal tissues of the elder population, a two- to three-fold increase in the expression of MAO-B has been observed, while the catalytic activity of this enzyme is elevated in the brains of AD patients, thus accelerating neurotransmitters’ consumption and neuronal damage.^28,29^

### 1.2 Binding Sites of Monoamine Oxidase A (MAO-A)

A thorough understanding of the binding site and its interactions with specific substrates or ligands is essential for enhancing the rational design of new MAO inhibitors, improving both their selectivity and effectiveness.

The binding site of MAO-A consists of a substrate cavity of about 550 Å^3^ in front of the FAD cofactor. The cavity formed by residues 210–216, extends from the FAD ring to the edge of itself.^30^ Due to the wide “aromatic cage”, the substrate cavity of MAO-A can accommodate relatively bulky aromatic groups. The major differences between the pockets of the two isoforms are Asn181, Phe208, and Ile335 in MAO-A, while Cys172, Ile199, and Tyr326 in MAO-B. In MAO-A, Ile335 occupies this position and produces a less pronounced restriction, when compared to the Tyr326 side chain in MAO-B, which does not directly divide the two cavities (Figure 2).^31^

**Figure 2:**
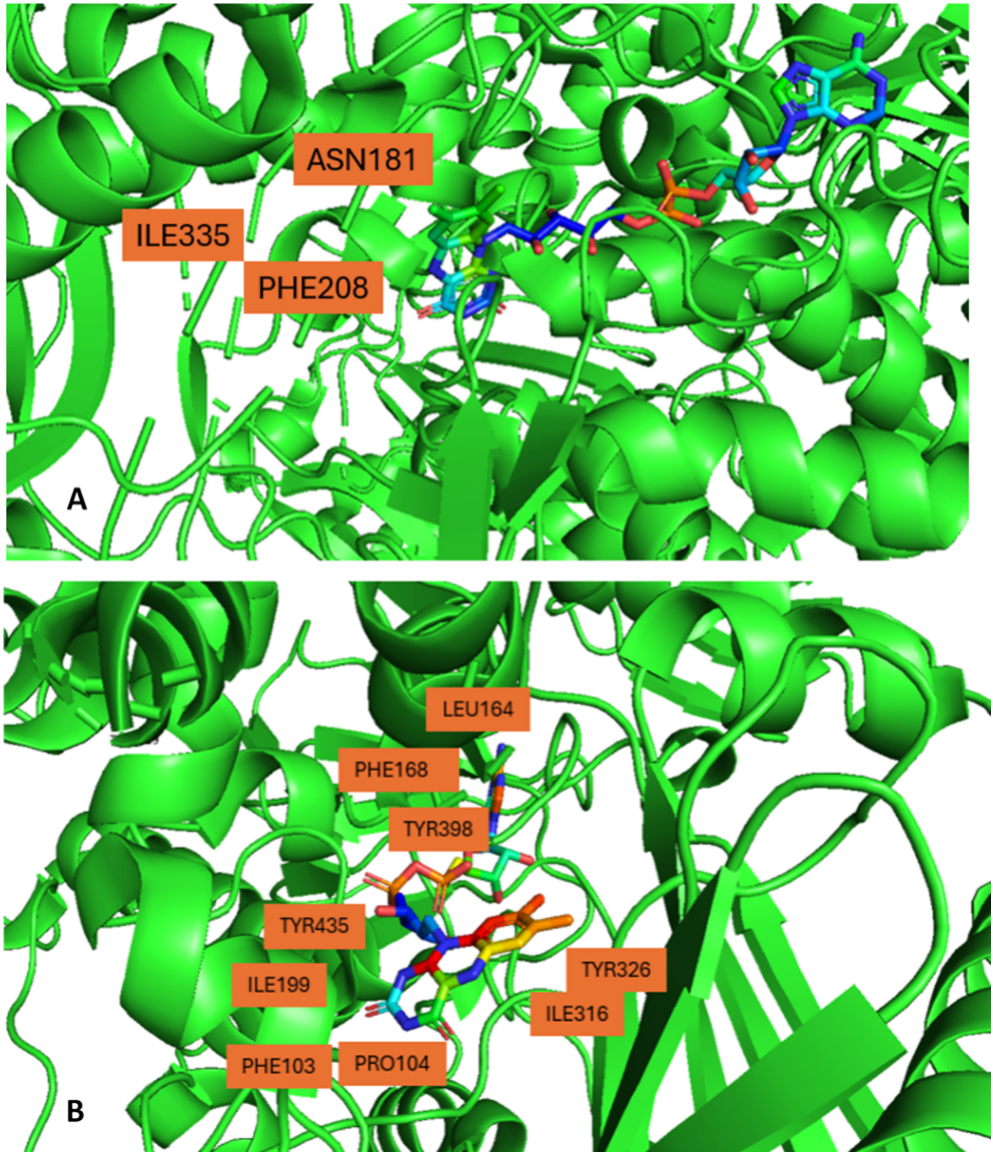
(A) Active Site of Monoamine Oxidase A from the pdb of 2BXR (*PyMOL*) (B) Active Site of Monoamine Oxidase B from the pdb of 1GOS *(PyMOL*).

### 1.3 Binding Sites of Monoamine Oxidase B (MAO-B)

Currently, there are four MAO-A crystal structures and more than 40 MAO-B crystal structures published in Protein Data Bank (PDB). The disclosure of monoamine oxidase crystal structures revealed the residues in the binding site of MAOs and also made structural information available for the rational design of novel MAOs’ inhibitors.

The binding site of MAO-B consists of a substrate cavity of about 420 Å^3^ and an entrance cavity of about 290 Å^3^. Combined, they form an elongated pocket with a volume of approximately 700 Å^3^.^32^ This pocket cavity originates from the isoalloxazine ring site of FAD and extends to the loop surface, and is filled with hydrophobic amino acid residues.^33^ Residues Tyr398 and Tyr435 are found to stack almost parallel to each other in front of the isoalloxazine ring and almost perpendicular to the plane of the FAD ring, thus generating an “aromatic cage” of the recognition site of the substrate amino group.^34^ A few highly conserved water molecules are located at the bottom of the inner cavity, creating a small hydrophilic region in front of FAD. This area may facilitate the binding of the amino group of the substrate being oxidized. (Figure 2).^33,35,36^ A smaller hydrophobic entrance cavity is located next to the substrate cavity, lined by the residues Phe103, Pro104, Trp119, Leu164, Leu167, Phe168, Leu171, Ile199, Ile316, and Tyr326. The side chains of Tyr326 and Ile199 play a crucial role in separating the two cavities.^37^

The primary conformational change in MAO-B occurs in the flexible Ile199 residue, whose side-chain position shifts based on the size of the ligand present in the active site. (Figure 2).^38^ Bulky ligands cause the Ile199 residue to adopt an open conformation, allowing the inner substrate cavity to be accessed from the surface of the loop.^35,38^ On the other hand, the dual-cavity active site is formed when small ligands occupy only the substrate cavity in front of FAD, with Ile199 in a closed conformation. Site-directed mutagenesis of Ile199Phe has shown that the reduced flexibility of the residues is due to the bulky Phe side chain, which narrows the entrance cavity.^33,38,39^ Hence, the flexibility of the catalytic site in MAO-B is governed by the conformation of the Ile199 residue.^33,37–39^

The single- and double-mutant forms of MAO-B, specifically the Ile199Ala and Ile199Ala, Tyr326Ala mutations have been thoroughly investigated, as the Ile199Ala mutation causes the entrance cavity of MAO-B to remain permanently open, while the double mutations of Ile199Ala and Tyr326Ala result in MAO-B-specific inhibitors exhibiting an affinity similar to that of MAO-A.^39–41^ This showcases the importance of amino acid residues Ile199 and Tyr326 as key determinants of substrate and inhibitor specificities, in MAO-B, while Phe208 and Ile335 are for MAO-A (Figure 2).^30,40,42^

### 1.4 Monoamine Oxidase Inhibitors

Monoamine oxidase inhibitors (MAOIs) can reduce the breakdown of monoamines, thereby protecting neurons from damage caused by neurotoxic byproducts. By inhibiting the enzymes’ catalytic activity, MAOIs lead to an increase in the levels of monoamine neurotransmitters (such as serotonin and dopamine) stored in the nerve terminals. MAOIs have been developed as therapeutic agents in two key categories, based on the substrate specificity and the distribution differences of the two enzyme isoforms in neuronal tissues.^43^ MAO-A selective inhibitors are used to treat mental disorders, such as anxiety and depression, whereas MAO-B selective inhibitors are primarily employed to manage neurodegenerative diseases like Alzheimer’s disease and Parkinson’s disease.^14,44,45^

In recent years, efforts have been made to develop effective compounds targeting MAOs, leading to the creation of various new chemical entities with promising properties. Typically, MAO inhibitors are categorized as selective or nonselective, reversible or irreversible inhibitors.

A lot of compounds have been synthesized for the inhibition of MAO enzymes. Aliphatic and aromatic amines, amides, anilides, hydrazines, hydrazones and hydrazides, pyrroles, pyrrolidines and pyrrolines are just a few of such examples.^12^ Also, coumarin derivatives, chalcones, pyrazoles and pyrazolones, natural products, propargylamines, etc. have been mentioned over the years by many research groups.^46^

### 1.5 General Synthetic Procedures of Propargylamine Inhibitors

There are many different ways and reaction pathways which can be utilized for the synthesis of propargylamine derivatives, both terminal and not. Here we present three common methods.

A very common method is the A^2^-coupling reaction which involves a ketone and an amine (Figure 3). It is a multicomponent reaction (MCR) that enables the synthesis of propargylamines through the coupling of a ketone, an amine, and a terminal alkyne. The reaction mechanism begins with the activation of the ketone, which reacts with the amine to form an imine intermediate. Simultaneously, the terminal alkyne is activated by metal catalysts such as copper (Cu), gold (Au), or silver (Ag). The activated alkynyl intermediate then undergoes nucleophilic addition to the imine, leading to the formation of the desired propargylamine. This reaction is particularly valuable for the synthesis of complex propargylamines with pharmaceutical applications, including MAO-A and MAO-B inhibitors, as well as other bioactive compounds.

**Figure 3:**
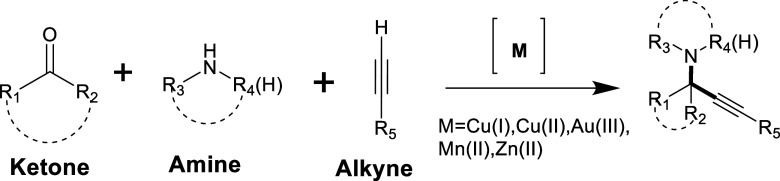
KA^2^ Coupling Reaction.

The A^3^-coupling reaction is a three-component reaction involving an aldehyde, an amine, and a terminal alkyne, leading to the formation of propargylamines (Figure 4). This reaction is catalyzed by metal complexes, most commonly copper (Cu), but also silver (Ag) or gold (Au) in some cases. The mechanism of the A^3^-coupling reaction begins with the condensation of the aldehyde and the amine to form an imine intermediate. Meanwhile, the terminal alkyne is activated by the metal catalyst, increasing its nucleophilicity. The activated alkynyl species then undergoes nucleophilic addition to the imine, resulting in the formation of the propargylamine. This reaction is widely used in organic synthesis due to its efficiency, mild reaction conditions, and atom economy. It plays a crucial role in the synthesis of biologically active molecules, including pharmaceutical compounds such as enzyme inhibitors, neurotransmitter analogs, and heterocyclic scaffolds.^47–53^

**Figure 4:**
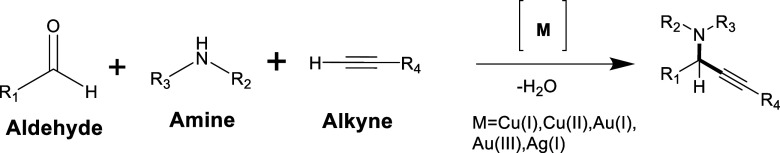
Generic A^3^ -coupling.

Propargylamines can also be synthesized through Copper-Catalyzed Retro-Mannich and Deselenative C–H Insertion Reactions (Figure 5). An intriguing method for synthesizing propargylamines via a retro-Mannich approach has been reported by Zhu et al. In this copper-catalyzed reaction, terminal alkyne derivatives react with Mannich bases, which are generated *in situ* through a chlorine­(1+) ion-triggered retro-Mannich-type fragmentation. The moiety of chlorine (1+) can be obtained, for example, from a reagent such as 1-chloro-2,5-dimethylenepyrrolidine. When a Mannich base (compound I) is treated with a terminal alkyne derivative in the presence of CuCl_2_, *N*-chlorosuccinimide (NCS), and NaHCO_3_, the desired propargylamines (II) are produced. The authors investigated different Mannich bases, and found that compound I provided propargylamines in higher yields. Several oxidants were tested, including NCS, NBS, I_2_, *^t^* BuOOH, and PhI­(OAc)_2_, with NCS providing the best conversion in the presence of NaHCO_3_. According to the proposed mechanism, Mannich base I is first chlorinated by NCS to form intermediate III. This then undergoes fragmentation, forming product IV and an iminium ion V. The phenylacetylene reacts with CuCl_2_·H_2_O, generating a copper acetylide intermediate through C–H activation. This intermediate subsequently reacts with the *in situ* formed iminium ion V to yield the propargylamine II.^50,54^

**Figure 5:**
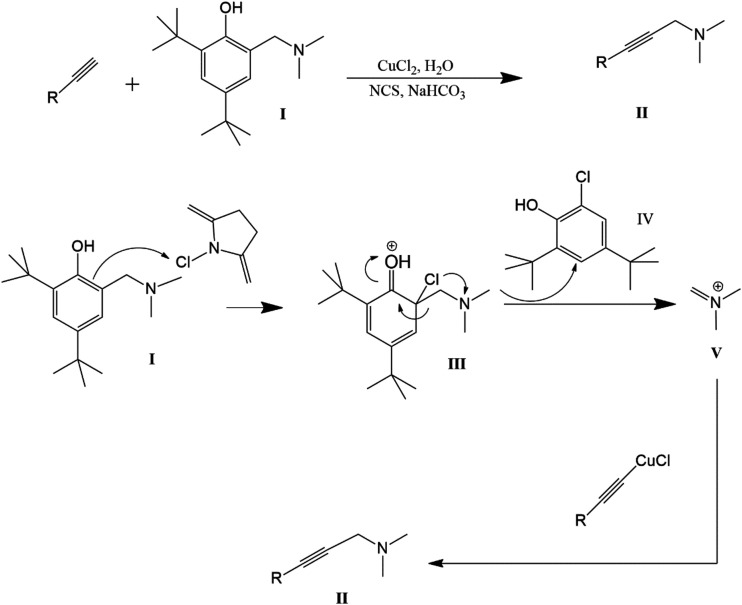
Copper-Catalyzed Retro-Mannich and Deselenative C–H Insertion Reaction.

There are many more synthetic pathways which can be followed for the synthesis of these molecules, but these are out of the scope of the current review.

### 1.6 Inhibitory Mechanism of Action of Propargylamines

The widely recognized mechanism of MAO inhibition by terminal alkyne-propargylamine-based drugs depends on their irreversible interaction with the target enzymes, thereby inhibiting their catalytic function.

In 2019, Tandarić and Vianello suggested a mechanism for the inhibition of MAO enzymes by rasagiline and selegiline, grounded in detailed theoretical calculations.^55^ According to this proposal, the FAD cofactor initially abstracts a hydride from the methylenic group of the propargylamine scaffold of the inhibitors, leading to the formation of I, which contains an allene moiety (Figure 6). This is followed by a proton transfer to the allenic carbon center, which forms a three-membered ring (II). The ring’s opening results in an irreversible drug-enzyme inhibition (III). The key element in the propargylamines’ success as MAO inhibitors is this irreversible binding to the enzyme. Also, in 2018, Albreht, Ramsay et al. studied the propargylamine-based ligand ASS234, which was suggested to undergo initial oxidation by MAO, resulting in the formation of the corresponding iminium cation and the enzyme FADH^–^ (Figure 6).^56,57^ A Michael addition of the flavin nitrogen to the electrophilic species to the MAO enzyme produced states V and VI, which deactivate its catalytic potential.^8,47,58,59^ In this review, some of the compounds discussed, do not follow this mechanism of inhibition, since they contain the propargylamine moiety in a nonterminal position. Thus, the proposed deactivation mechanism must follow a different pathway.

**Figure 6:**
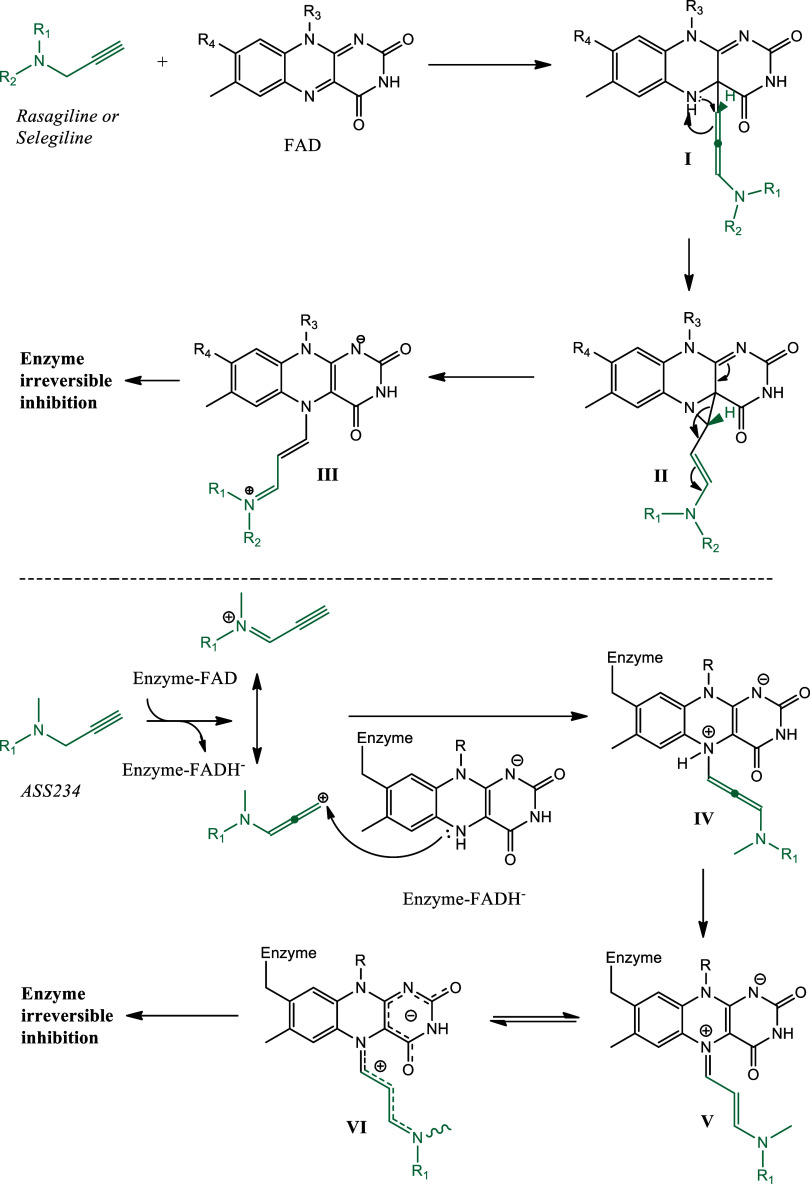
Proposed inhibition mechanism of action for rasagiline/selegiline and propargylamine ASS234.

Other mechanistic pathways for propargylamines in MAO enzyme inhibition, involve a redox or nucleophilic substitution process. The pathways include Single Electron Transfer (SET), Polar Nucleophilic, Hydride, and Two-Step Hydride mechanisms, each leading to the final product. Figure 7 uses color-coded molecular structures to highlight different functional groups and reaction intermediates. The pathways involve electron transfers, nucleophilic attacks, and hydride shifts, ultimately resulting in the cleavage of a molecular bond and the formation of a final product and a byproduct. The diagram effectively compares the mechanistic variations and their respective reaction intermediates.^8^

**Figure 7:**
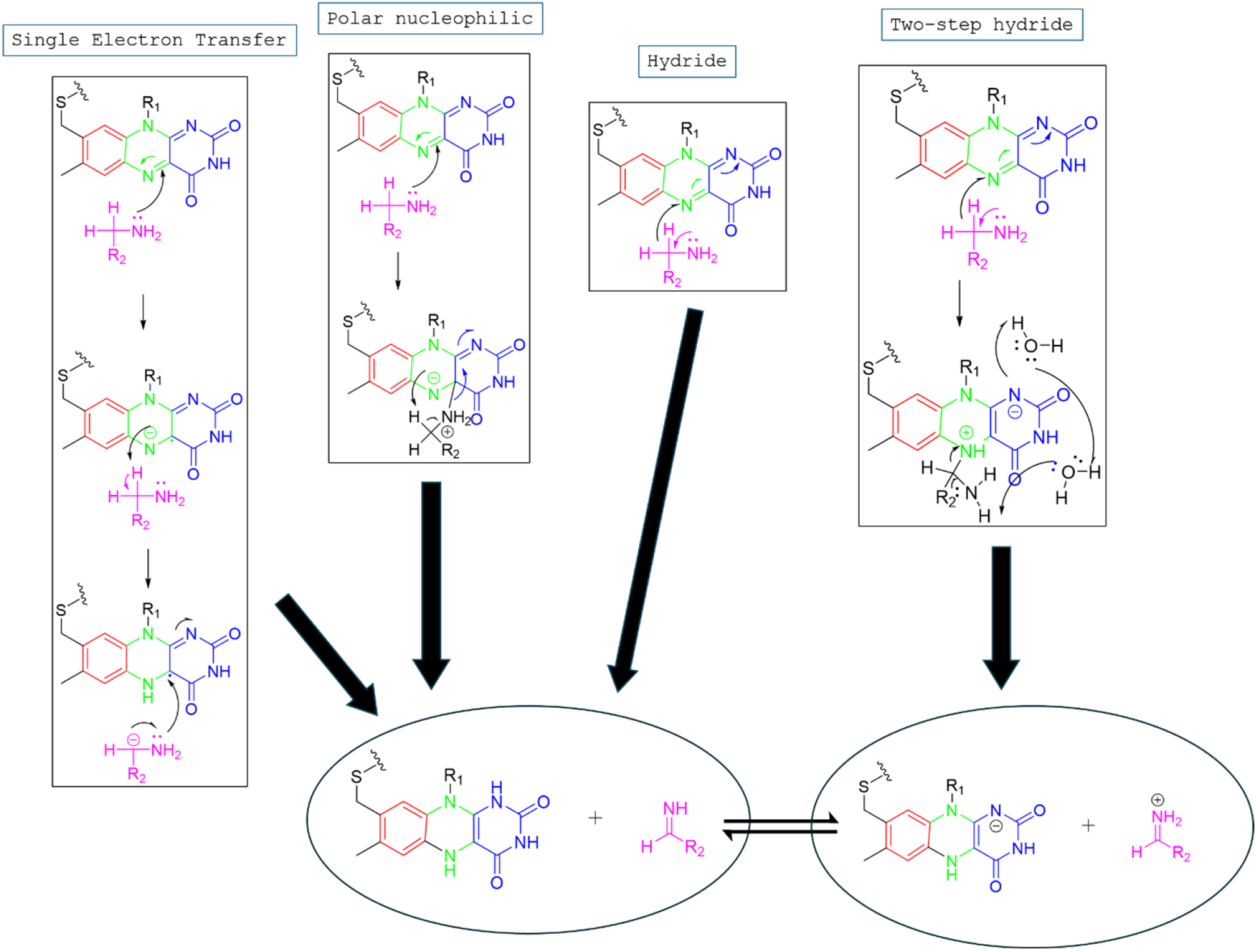
Proposed inhibition mechanism of action via Single Electron Transfer (SET), Polar Nucleophilic, Hydride, and Two-Step Hydride mechanistic pathways.

It is well-known that via these irreversible mechanisms of inhibition, a lot of patients develop many side effects. A major and potentially life-threatening side effect of MAOIs is the hypertensive crisis, commonly referred to as the “cheese effect.” This occurs when MAOIs are combined with sympathomimetic amines like tyramine, which is present in certain fermented foods such as cheese. It is worth noting that selective MAO-B inhibitors do not cause this side effect because MAO-B is present in very low amounts in the intestines.^60,61^ Since the inhibition of hMAO-B offers the anti-Alzheimer’s effects desired, selective inhibition of this isoform is desirable.

Moreover, while most of the terminal propargylamines agree with these mechanisms of action, via covalent bonding to the active site of the enzyme, not all of them showcase an irreversible mechanism of action. Many of the inhibitors listed below in this review and possess a terminal propargylamine group, are reversible inhibitors for hMAO-B. Moreover, this review emphasizes the importance of the development of novel molecules with an internal propargylamine moiety, which in no way can it covalently bind to the MAO enzymes, thus avoiding the negative consequences resulting from the irreversible inhibition of this enzyme. Typical examples on the synthesis and biological action of such molecules are the works of Kulikova et al. and Mavroeidi et al. which are discussed later in this review.

## 2 Drugs That Inhibit hMAO-B and Their Potential Use in Alzheimer’s Disease

Certain highly effective MAO inhibitors, such as rasagiline, selegiline, and clorgyline, feature an N-propargyl group, which is believed to be the crucial pharmacophore responsible for inhibiting MAO through a covalent interaction with the FAD unit.^62^

Rasagiline (Figure 8), primarily known for its use in treating Parkinson’s disease, has also been studied for its potential benefits in Alzheimer’s disease, particularly in relation to its effects on monoamine oxidase B. For rasagiline, its IC_50_ value in inhibiting MAO-B is typically reported around 0.03 μM. This reflects its potency as a MAO-B inhibitor, with rasagiline demonstrating a strong affinity for this enzyme. This value can vary slightly, based on the experimental conditions and the specific assay used, but the 0.03 μM range is commonly cited in pharmacological studies.^20,63,64^

**Figure 8:**
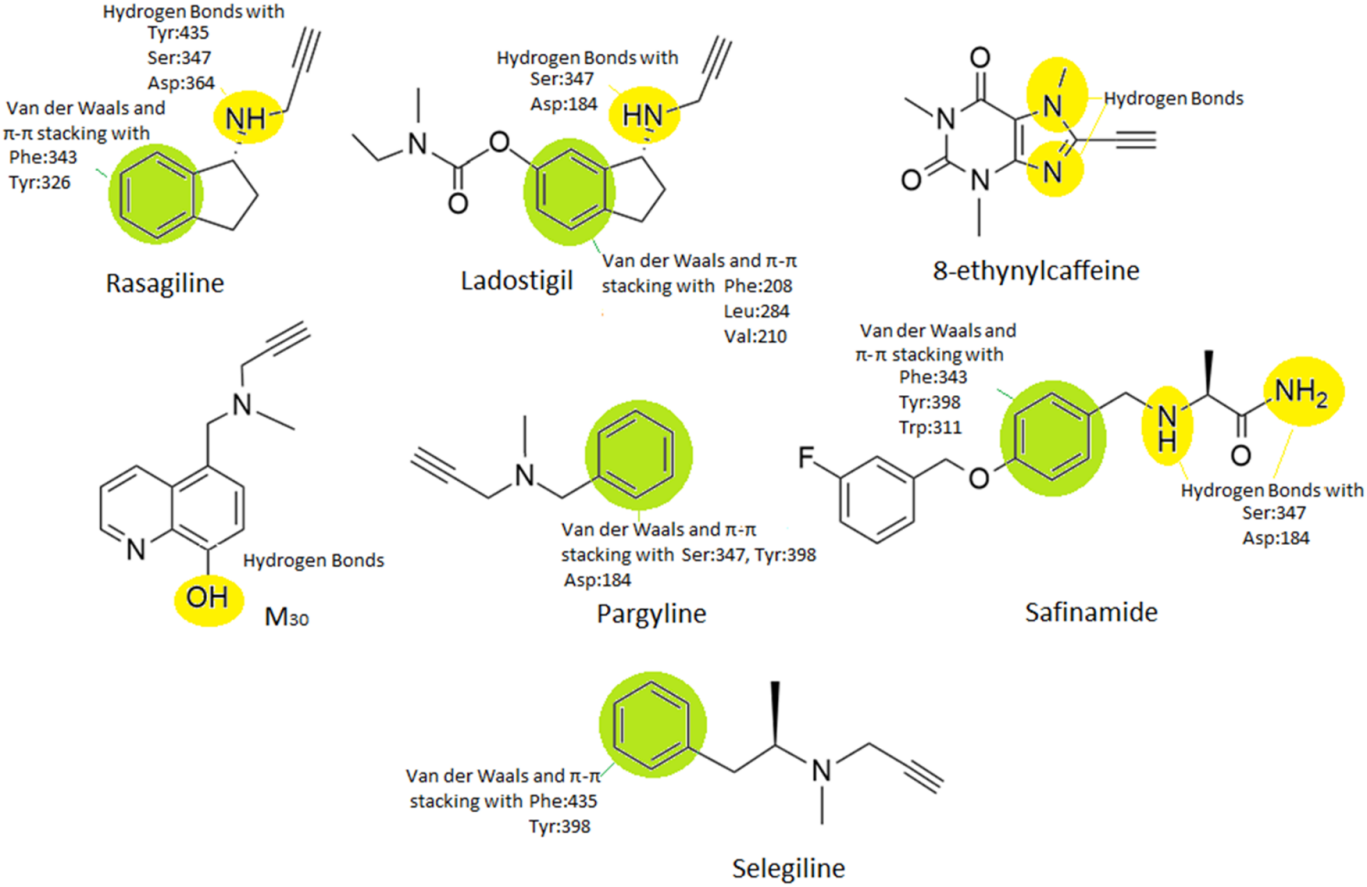
Structure–activity relationships (SARs) with hMAO-B for the structures of rasagiline, ladostigil, 8-ethynylcaffeine, M30, pargyline, safinamide and selegiline are highlighted. All seven shown compounds are highlighted with their important pharmacophore segments. With green color is shown aromatic significance that exerts van der Waals and π–π stacking. The yellow color molecular segment shows their participation in hydrogen bonding.

Selegiline (Figure 8), chemically known as (*R*)-*N*,α-dimethyl-1-phenylpropan-2-amine, is most commonly used for Parkinson’s disease and depression, while its role in Alzheimer’s disease remains an area of interest in clinical research. Its potential role in Alzheimer’s disease has been investigated, particularly due to its neuroprotective properties and effects on dopamine and other neurotransmitters in the brain. It has an IC_50_ value of approximately 5–10 nM with regards to MAO-B inhibition, depending on the specific assay or experimental conditions used. In Alzheimer’s disease, there is a significant loss of dopamine, and the idea is that by inhibiting the breakdown of dopamine, selegiline might help preserve dopamine levels, potentially improving cognitive symptoms or slowing cognitive decline. In addition, there is evidence that selegiline has antioxidant properties, which could help protect neurons from oxidative stress—one of the factors believed to contribute to Alzheimer’s pathology. Early studies on selegiline in Alzheimer’s disease suggested that it could offer modest improvements in cognition and global functioning, particularly in patients with mild to moderate Alzheimer’s. However, these results were somewhat inconsistent and did not demonstrate significant disease-modifying effects. Moreover, selegiline has been tested in combination with other treatments for Alzheimer’s, such as cholinesterase inhibitors (e.g., donepezil). The hypothesis is that selegiline’s neuroprotective effects may complement the action of these drugs, which focus on increasing acetylcholine levels. Some studies have explored the idea that selegiline may help delay the progression of Alzheimer’s disease by offering neuroprotection. This could theoretically slow the rate of neuronal degeneration in the brain, but more robust clinical evidence is needed to confirm this effect. A number of studies have continued to investigate selegiline in the context of Alzheimer’s disease. The results are still mixed, with some studies showing small cognitive benefits and others finding no significant effects. In one study, the combination of selegiline with donepezil was shown to have some benefit in terms of cognitive function, but the improvements were modest and did not translate to significant long-term benefits. The administration of selegiline for Alzheimer’s disease has generally followed the same approach as used for Parkinson’s disease, although there is a lack of consensus on the optimal dose and treatment duration for Alzheimer’s. As of recent clinical guidelines, selegiline is not considered a first-line treatment for Alzheimer’s disease. It may be considered in specific cases, particularly when other treatments are insufficient or not tolerated. Its primary role remains adjunctive, rather than as a primary therapeutic agent for cognitive improvement.^63,65–70^

Due to its propargylamine structure, pargyline (Figure 8) has also been studied in the context of neurodegenerative diseases, like Alzheimer’s disease. The IC_50_ of pargyline for MAO-B inhibition is generally reported to be in the low micromolar range (around 1–2 μM). While pargyline and its propargylamine structure show potential for neuroprotection and increased neurotransmitter levels, its role in Alzheimer’s treatment remains experimental and would need more focused research before it could be considered a standard therapy for the disease. Other MAO-B inhibitors or alternative treatments are currently more common for Alzheimer’s management.^22^

Ladostigil (Figure 8) is an experimental drug being studied for its potential use in treating Alzheimer’s disease. It is a dual inhibitor of both acetylcholinesterase (AChE), thus increasing the levels of acetylcholine, a neurotransmitter that is deficient in Alzheimer’s patients, and monoamine oxidase B (MAO-B). Both these enzymes are involved in the degradation of important neurotransmitters in the brain. By inhibiting these enzymes, ladostigil is thought to help improve the availability of neurotransmitters such as acetylcholine, dopamine, and serotonin, which are important for cognitive function and mood regulation. The IC_50_ for MAO-A inhibition by ladostigil is reported to be approximately 27 nM, while for MAO-B is about 23 nM.^22,64,71^

The use of safinamide (Figure 8) and its relationship with Alzheimer’s disease is a topic of growing interest. Safinamide is primarily approved for Parkinson’s disease, but its potential for treating Alzheimer’s disease is also being explored, particularly due to its mechanism involving monoamine oxidase B inhibition and modulation of glutamate release. Safinamide’s IC_50_ value for MAO-B inhibition is typically in the low micromolar range, meaning that it is a potent inhibitor of MAO-B. Based on various studies, the IC_50_ of safinamide for MAO-B inhibition is usually reported to be around 10 to 100 nM. While it is primarily marketed for Parkinson’s disease, there is interest in its potential for Alzheimer’s disease due to its MAO-B inhibition and glutamate modulation. This compound does not contain a propargylamine group and inhibits MAO-B through a reversible mechanism.^63^

M30 (Figure 8) is a propylamine-like compound associated with research in Alzheimer’s disease and other neurodegenerative conditions. The name “M30” refers to a specific molecule, and “propylamine” refers to a chemical group containing a propyl moiety (C_3_H_7_) attached to an amine group (NH_2_). In the context of Alzheimer’s, M30 is not as widely known as some other treatments, but it could be a candidate in experimental or preclinical research stages. M30 is a compound that has been studied for its effects on enzymes involved in neurodegenerative diseases like Alzheimer’s, specifically monoamine oxidase (IC_50_ = 57 nM for MAO-B) and acetylcholinesterase.^22^

8-Ethynylcaffeine (Figure 8) is a synthetic analog of caffeine, developed by modifying the structure of caffeine with an ethynyl group attached at the 8-position of the purine ring. This modification is aimed at exploring new bioactive properties or enhancing certain pharmacological effects in treating neurological disorders, such as Parkinson’s and Alzheimer’s disease (MAO-B; IC_50_: 101 ± 1.7 nM). Its caffeine-like effects on the central nervous system might also make it a candidate for further investigation as a cognitive enhancer or stimulant.^72^

The structure–activity relationship (SAR) analysis of the seven compounds depicted in Figure 8 shows key molecular features contributing to their inhibitory potency against human monoamine oxidase B (hMAO-B). Among the compounds, rasagiline exhibits very good potency (IC_50_ = 30 nM), which correlates with its ability to form multiple hydrogen bonds (with Tyr435, Ser347, and Asp364) and engage in π–π stacking and van der Waals interactions with aromatic residues (Phe343 and Tyr326) in the enzyme’s active site. Selegiline (IC_50_ = 5–10 nM) shows stronger inhibition, primarily through π–π stacking with Phe435 and Tyr398, although it lacks the extensive hydrogen bonding network seen in rasagiline. Safinamide, with an IC_50_ ranging from 10–100 nM, benefits from both hydrogen bonding and π–π interactions, but its flexible side chain may result in less optimal binding. Ladostigil (IC_50_ = 23 nM) and M30 (IC_50_ = 57 nM) exhibit moderate potency, with M30 relying mainly on hydrogen bonding via its hydroxyl group and lacking significant aromatic stacking. 8-ethynylcaffeine (IC_50_ = 101 nM) shows limited hydrogen bonding and π–π stacking, which may explain its reduced efficacy. Finally, pargyline, with the weakest activity (IC_50_ = 2 μM), demonstrates minimal interaction with the binding site, reflecting its limited hydrogen bonding and simple structure. Overall, the most potent inhibitors combine strong hydrogen bonding capabilities with π–π stacking interactions, highlighting these as crucial elements for effective MAO-B inhibition.

## 3 Novel Propargylamine Derivatives for the Treatment of Alzheimer’s Disease 2020–2024

A bibliographic search was carried out concerning all molecules that were synthesized from 2020 to 2024 containing within their structure the propargylamine moiety and inhibiting hMAO-B. The compounds studied in this review were selected based on their biological activity *in vitro* and/or *in vivo* experiments. Thus, compounds that present the lowest IC_50_ values were selected to be presented, from each article. Structure–Activity Relationship (SAR) studies were also conducted and the types of interactions between the various pharmacophore groups of the inhibitors and the respective amino acids are outlined as well.

### 3.1 2020

Guieu et al. designed and synthesized a compound containing the structures of donepezil (DPZ) and rasagiline, two indane derivatives marketed as AChE and MAO-B inhibitors, respectively, which they called propargylaminodonepezil (PADPZ) (Figure 9). The synthesis of racemic trans-PADPZ was achieved and its biological evaluation established its inhibitory activities toward both hAChE (IC_50_ = 0.442 ± 0.03 μM) and hMAO-B (IC_50_ = 6.43 ± 0.62 μM) when compared with reference compounds rasagiline (hMAO-B; IC_50_ = 0.014 μM) and pargyline (hMAO-B; IC_50_ = 2.69 ± 0.48 μM).^73^ This compound can be characterized as a good lead-compound, which needs further optimization. From SAR studies it is revealed that residue Tyr326 interacts with the benzyl- ring of the methoxybenzyl- group of *trans*-PADPZ (Figure 9). Specifically, van der Waals and π–π stacking interactions are developed between the residue and the compound. Furthermore, residue Gln206 develops hydrogen bonds with the oxygen of the amide group of *trans*-PADPZ via the contribution of a water molecule. Lipophilic interactions, also, take place between the compound and the nearby amino acids inside the active site of the enzyme.

**Figure 9:**
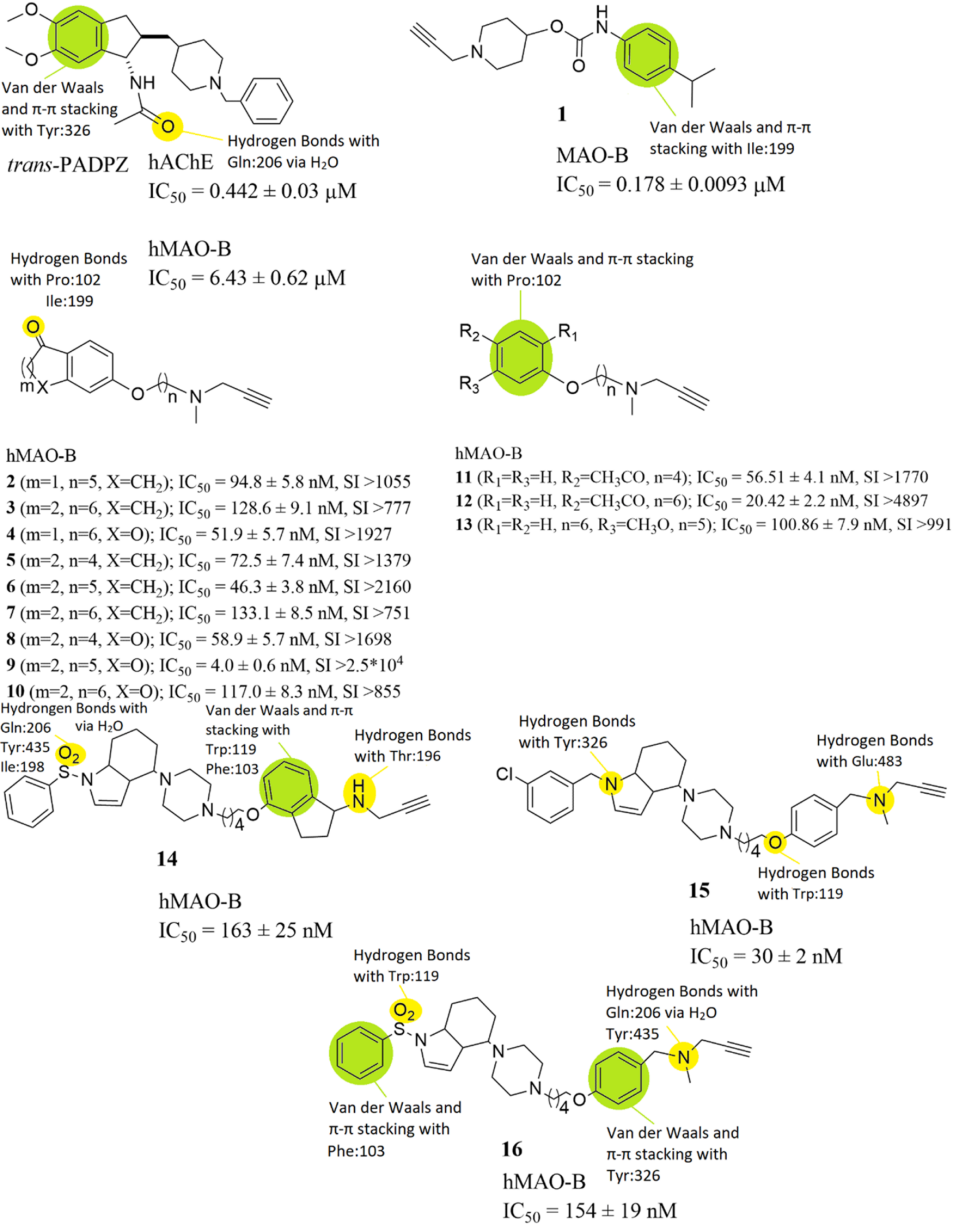
Structures of compounds *trans-*PADPZ and **1** through **16** and their half maximal inhibitory concentrations (IC_50_). The structure–activity relationships (SARs) of these molecules with hMAO-B are highlighted as well.

A series of 36 new *N*-alkylpiperidine carbamates was designed, synthesized and evaluated by Košak et al. as multitarget directed ligands (MTDLs), inhibiting MAO-A and MAO-B as well. Between them, selective MAO-B inhibitor compound **1** (Figure 9), showed the lowest IC_50_ value of 0.178 ± 0.0093 μM when compared with the reference, rasagiline (hMAO-B; IC_50_ = 0.036 ± 0.004 μM). Results of enzyme kinetics experiments showed that compound **1** is an irreversible and time-dependent inhibitor of MAO-B, which also prevents amyloid β1–42 (Aβ1–42)-induced neuronal cell death. Moreover, the results from the PAMPA-BBB assay indicate that this compound should cross the blood–brain-barrier (BBB). The neuroprotective effects of compound **1** could be the result of its Aβ1–42 antiaggregation effects.^74^ From SAR studies it is revealed that residue Ile199 develops van der Waals and π–π stacking interactions with the phenyl-group of compound **1** (Figure 9). Lipophilic interactions, also, take place between the compound and the nearby amino acids inside the active site of the enzyme.

Xie et al. designed, synthesized and evaluated a series of rasagiline-clorgyline hybrids. All the target compounds were investigated for their ability to inhibit the monoamine oxidases and amyloid-β aggregation. All compounds (**2–13**) were selective hMAO-B inhibitors with IC_50_ values ranging from 133 nM to 4 nM, and could penetrate the blood–brain barrier (Figure 9). Among these compounds, compound **9** exhibited higher hMAO-B potency and selectivity [IC_50_ = 4.0 ± 0.6 nM, SI (Selective Index) for hMAO-B = IC_50_ (hMAO-A)/IC_50_ (hMAO-B) > 25,000] than the reference inhibitor rasagiline (IC_50_ = 141.70 ± 6.34 nM, SI for hMAO-B > 355), as well as good inhibition of Aβ1–42 aggregation. In addition, kinetic and molecular modeling studies suggested compound **9** was a competitive and reversible inhibitor for hMAO-B. Meanwhile, compound **9** showed low cytotoxicity and neuroprotective effects, according to cell viability and neuroprotection activity assays. Further pharmacokinetics studies showed that compound **9** had good pharmacokinetic characteristics, after intravenous and oral administrations. These properties highlighted that compound **9** could serve as an effective and promising candidate for AD therapy.^75^ From SAR studies it is revealed that residues Ile199 and Pro102 develop hydrogen bonds with the oxygen atoms of the carbonyl- groups of compounds **2**-**10** (Figure 9). On the other hand, compounds **11**-**13** showcase van der Waals and π–π stacking interactions via their phenyl- groups with amino acid Pro102. Lipophilic interactions, also, take place between all of these compounds and the nearby amino acids inside the active site of the enzyme.

Canale et al. designed, synthesized and evaluated compounds integrating the (indol-4-yl)­piperazine moiety and frameworks of known MAO-B inhibitors (aryloxy fragments), connected through an alkylene linker. These molecules were designed as multitarget directed ligands (MTDLs) and inhibit MAOs. Compounds **14** (IC_50_ = 163  ± 25 nM), **15** (IC_50_ = 30  ± 2 nM) and **16** (IC_50_ = 154  ± 19 nM) had the best inhibitory activity against hMAO-B, with compound **15** being the most potent (Figure 9). The reference compound used was rasagiline with (hMAO-B; IC_50_ = 15.4  ±  0.6 nM). Nevertheless, compound **16** had the most balanced pharmacologic profile and was used for further examinations. Compound **16** was metabolically stable and brain penetrant, while it reduced the gliotoxic effect of 6-OHDA in C8-D1A astrocytes in two complementary assays (MTT and LDH) assessing cell survival. Most importantly, this effect was not observed after treatment with MAO-B inhibitor selegiline. Finally, compound **16** also reversed cognitive decline induced by scopolamine in the NOR test in rats, making it an interesting prototype in the development of new treatment strategies for AD.^76^ From SAR studies it is revealed that residues Gln206, Tyr435 and Ile198 develop hydrogen bonding interactions with the oxygen atom of the sulfoxide- group of compound **14** (Figure 9). Hydrogen bonding interactions are also developed between the amine- group of the compound and Thr196. Moreover, van der Waals and π–π stacking interactions are established between the benzyl- ring of the indane- group of the compound and amino acids Trp119 and Phe103. Compound **15** develops hydrogen bond interactions with Tyr326 via the nitrogen atom of the polycyclic- group in its structure, with Trp119 via the oxygen atom in its structure and with Glu483 via the nitrogen atom of the propargylamine- group in its structure (Figure 9). Finally, compound **16** shows hydrogen bond interactions with Trp119 via the oxygen atom of the sulfoxide- group in its structure and with Gln206 and Tyr435 via the nitrogen atom of the propargylamine- group in its structure with the participation of a water molecule (Figure 9). Van der Waals and π–π stacking interactions are developed as well between the phenyl- group near the sulfoxide- moiety and Phe103 and the phenyl- group near the propargylamine- moiety and Tyr326. Lipophilic interactions, take place between all of these compounds and the nearby amino acids inside the active site of the enzyme.

Li et al. designed and synthesized a series of pyridoxine-resveratrol hybrids as monoamine oxidase B inhibitors for the treatment of Parkinson’s disease (PD). Since some of these molecules were excellent selective inhibitors of hMAO-B, they could also be used for the treatment of Alzheimer’s disease. Specifically, compound **17** (Figure 10) showed the most excellent inhibition to hMAO-B with an IC_50_ value of 0.01 ± 0.005 μM when compared with reference compounds rasagiline (hMAO-B; IC_50_ = 0.0437 ± 0.002 μM) and clorgiline (hMAO-B; IC_50_ = 8.85 ± 0.201 μM). Further reversibility studies demonstrated that **17** was an irreversible MAO-B inhibitor. In addition, this compound also exhibited low cytotoxicity and excellent neuroprotective effect in the test on H_2_O_2_-induced PC-12 cell injury. Moreover, **17** showed good antioxidant activity and high blood–brain barrier permeability. Overall, all of these results highlighted **17** as a potential and excellent MAO-B inhibitor for PD and AD treatment.^77^ From SAR studies it is revealed that residues Leu171 and Ile198 develop van der Waals interactions with the phenyl- group of compound **17** (Figure 10). Lipophilic interactions, also, take place between the compound and the nearby amino acids inside the active site of the enzyme.

**Figure 10:**
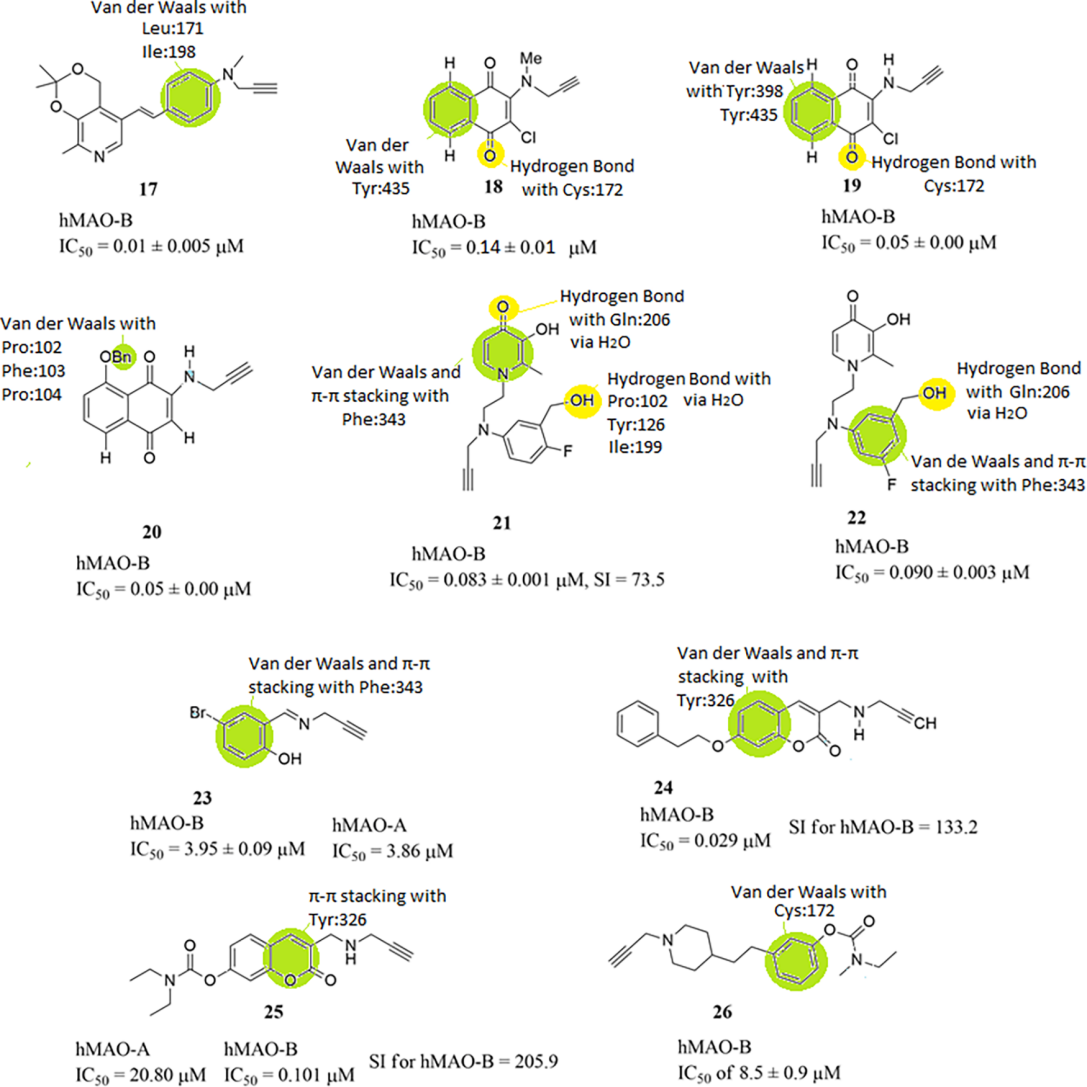
Structures of compounds **17** through **26** and their half maximal inhibitory concentrations (IC_50_). The structure–activity relationships (SARs) of these molecules with hMAO-B are highlighted as well.

### 3.2 2021

Mezeiova et al. constructed several N-propargylamine-NQ derivatives as MTDLs. These N-PNQs and N-(M)­PNQs were evaluated for their ability to inhibit Aβ aggregation and MAOs. From the tested compounds, the best inhibitory activity for the inhibition of hMAO-B was shown by compounds **18** (IC_50_ 0.14 ± 0.01 μM), **19** (IC_50_ 0.05 ± 0.00 μM) and **20** (IC_50_ 0.05 ± 0.00 μM) (Figure 10) when compared with reference compound pargyline (hMAO-B; IC_50_ = 0.08 ± 0.01 μM). In particular, derivatives **18** and **19**, which contain a chlorine atom at position 2, demonstrated strong MAO-B inhibition and metal-chelating properties for Cu­(II), with compound **19** having the strongest inhibitory activity and being a specific inhibitor for MAO-B, when compared to the inhibitory activity for hMAO-A (IC_50_ 7.76 ± 0.61 μM, SI for hMAO-B = 155). Moreover, **18** showed satisfactory inhibition activity against Aβ aggregation (40.5 ± 6.7%). Compound **20**, bearing a benzyloxy group, was the only one demonstrating anti-inflammatory effects at concentrations corresponding to 1.0 or 0.5 MTC in SIM-A9 cell line, and showed more profound radical scavenging activity in the presence of Cu­(II) ions than it was observed for standard clioquinol. These findings warrant further investigation of N-PNQs and N-(M)­PNQs as disease-modifying agents in AD treatment.^21^ From SAR studies it is revealed that residue Cys172 develops hydrogen bonds with the oxygen atom of the 1,4-naphthoquinone- group of compound **18** (Figure 10). Moreover, van der Waals interactions are developed between Tyr435 and the benzyl- ring of the 1,4-naphthoquinone- group of compound **18**. For compound **19** (Figure 10) the same type of interactions are observed, with an added van der Waals interaction between Tyr398 and the benzyl- ring of the 1,4-naphthoquinone- group. Furthermore, van der Waals interactions are observed between the benzyl ether- group of compound **20** and amino acid residues Pro102, Phe103 and Pro104 (Figure 10). Lipophilic interactions, take place between all of these compounds and the nearby amino acids inside the active site of the enzyme.

Guo et al. rationally designed twenty-nine hybrids of *N*-propargylamine-hydroxypyridinone based on the MTDL drug design and pharmacophore fusion strategy. These hybrids displayed promising iron-chelating activity and potent MAO-B inhibitory activity in the *in vitro* tests. The hMAO-B inhibitory abilities of compounds **21** (IC_50_ = 0.083 ± 0.001 μM) and **22** (IC_50_ = 0.090 ± 0.003 μM) were the most potent compared to pargyline (IC_50_ = 0.097 ± 0.004 μM)), which was used as reference (Figure 10). Compound **21** also showed excellent selectivity for hMAO-B (hMAO-A IC_50_ = 6.11 ± 0.08 μM; SI for hMAO-B = 73.5). More importantly, the BBB permeability of **21** was predicted on multiple platforms (ADMETlab, SwissADME, and admetSAR), and all of the results displayed favorable BBB permeability. In the mouse Morris water maze model, **21** significantly ameliorated the cognitive dysfunction induced by scopolamine through behavioral evaluation. Overall, compound **21** is a multitarget hybrid with potential anti-AD activity.^22^ From SAR studies it is revealed that the oxygen atom of the carbonyl- group of compound **21** forms hydrogen bonds with residue Gln206 via a water molecule, while the hydroxyl- group on the benzyl- ring forms hydrogen bonds with residues Pro102, Tyr126 and Ile199 via the contribution of a water molecule (Figure 10). Van der Waals and π–π stacking interactions are also developed between the pyridine- ring and Phe343. For compound **22** the same types of interactions are observed, except the hydrogen bonds with Pro102, Tyr126 and Ile199, while the van der Waals and π–π stacking interactions are observed between the phenyl- group and Phe343 (Figure 10). Lipophilic interactions, also, take place for both of these compounds and the nearby amino acids inside the active site of the enzyme.

New propargylamine substituted derivatives combined with salicylic and cinnamic scaffolds were designed and synthesized by Krátký et al. as potential MTDLs, which also inhibit monoamine oxidases. 4-Bromo-2-[(prop-2-yn-1-ylimino)­methyl]­phenol **23** (Figure 10) was the most potent inhibitor of hMAO-B (IC_50_ of 3.95 ± 0.09 μM) along with a balanced inhibition of the other targets, being a real MTDL. The reference compound used was selegiline (hMAO-B; IC_50_ = 32.98 ± 0.08 nM). *In silico* prediction of physicochemical parameters affirm that the molecules would be active after oral administration and able to reach brain tissue.^78^ From SAR studies it is revealed that van der Waals and π–π stacking interactions between the phenyl- group of compound **23** and Phe343 are developed (Figure 10). Lipophilic interactions, also, take place between the compound and the nearby amino acids inside the active site of the enzyme.

Mzezewa et al. designed, synthesized, and evaluated 3,7-substituted coumarin derivatives as multifunctional Alzheimer’s disease agents. Compounds **24** (hMAO-A; IC_50_ = 3.86 μM and hMAO-B; IC_50_ = 0.029 μM with SI for hMAO-B = 133.2) and **25** (hMAO-A; IC_50_ = 20.80 μM and hMAO-B; IC_50_ = 0.101 μM, SI for hMAO-B = 205.9) (Figure 10) had the best selectivity for hMAO-B, while the best inhibitory activity for hMAO-B possessed compound **24**. Selegiline was used as reference (hMAO-B; IC_50_ = 0.010 μM). The time-dependent inhibition of MAO-B by the most promising and selective MAO-B inhibitors namely **24** and **25**, was also evaluated. Both **24** and **25** can be considered as reversible inhibitors of MAO-B taking into account that the propargylamine function does not bind to the FAD cofactor covalently, as is the case in selegiline. SH-SY5Y neuroblastoma cells treated with neurotoxin 1-methyl-4-phenylpyridinium (MPP+), which induces an apoptotic cascade in neurons, and compounds **24** and **25** had 92 and 85% survival rates respectively, compared to the MPP+ only treated ones, further demonstrating the moiety’s significance in neuroprotection. Unfortunately, the compounds, at all concentrations, did not show any significant ability to mitigate the cytotoxic effects caused by Aβ 25–35 peptide fragment, which is used to induce neurotoxicity in SH-SY5Y cells. Both **24** and **25** have high predicted intestinal absorption and BBB permeability, after an *in silico* pharmacokinetic and drug-likeness evaluation, using SWISSADME. All these properties of **24** and **25** make them good candidates for further testing for the treatment of Alzheimer’s disease.^79^ From SAR studies it is revealed that van der Waals and π–π stacking interactions between the phenyl- moiety of the α-benzopyrone- group of compound **24** and Tyr326 are developed, while π–π stacking interactions are observed between the pryran- moiety of the α-benzopyrone- group of compound **25** and Tyr326 (Figure 10). Lipophilic interactions take place for both of these compounds and the nearby amino acids inside the active site of the enzyme.

Mazej et al. synthesized 4-phenethyl-1-propargylpiperidine derivatives as MTDLs, which also inhibit hMAO-B. Compound **26** (Figure 10) exhibits low micromolar inhibition of hMAO-B (IC_50_ of 8.5 ± 0.9 μM) and a balanced pharmacologic profile. The docking studies and time-dependent inhibition of hBChE confirmed the initial expectation that the introduced carbamate moiety is responsible for covalent inhibition. Thus, dual-acting compound **26** serves as an excellent foundation for the further optimization of balanced MTDLs.^80^ From SAR studies it is revealed that van der Waals interactions between the phenyl- group of compound **26** and Cys172 are developed (Figure 10). Lipophilic interactions, also, take place between this compound and the nearby amino acids inside the active site of the enzyme.

Nadeem et al. designed and evaluated a series of fluoxetine and sertraline hybrids as multitarget inhibitors, which also inhibit hMAO-B. Compounds **27**, **28** and **29–31** (Figure 11) possess excellent concomitant inhibitory activity against hMAO-A/B enzymes as well as another target and thus emerged as optimal multitarget hybrids. Among them the best molecule containing a propargylamine moiety was compound **28**, which inhibits hMAO-B with an IC_50_ value of 0.015 ± 0.001 μM. Safinamide was the reference used (hMAO-B; IC_50_ = 0.025 ± 0.003 μM). In the acute toxicity studies of compound **28** there were no mortalities and no behavioral changes noticed in the experimental mice. Based on the observation in the acute toxicity result, a dose of 2000 mg/kg was measured as the safe dose.^81^ From SAR studies it is revealed that hydrogen bonds are developed between the oxygen atoms of the carbonyl- groups of compounds **27** and **28** with the amino acid residues Ile199 and Pro102 with the contribution of a water molecule. van der Waals and π–π stacking interactions are also observed between Phe118 and the phenyl- groups of compounds **27** and **29** and between His115 and His90 and the phenyl- moieties of the trifluorotoluene- groups of compounds **27** and **28** (Figure 11). The same type of interactions are observed between Phe103 and the phenyl- moieties of the benzyl ether- groups closer to the trifluorotoluene- groups of compounds **27** and **28**, while they are observed as well with the naphthalene- moiety of compound **31** (Figure 11). Finally, van der Waals and π–π stacking interactions are developed between the phenyl- moiety of the tetralin- group of compound **30** and residue Phe168 (Figure 11). Lipophilic interactions take place between all of these compounds and the nearby amino acids inside the active site of the enzyme.

**Figure 11:**
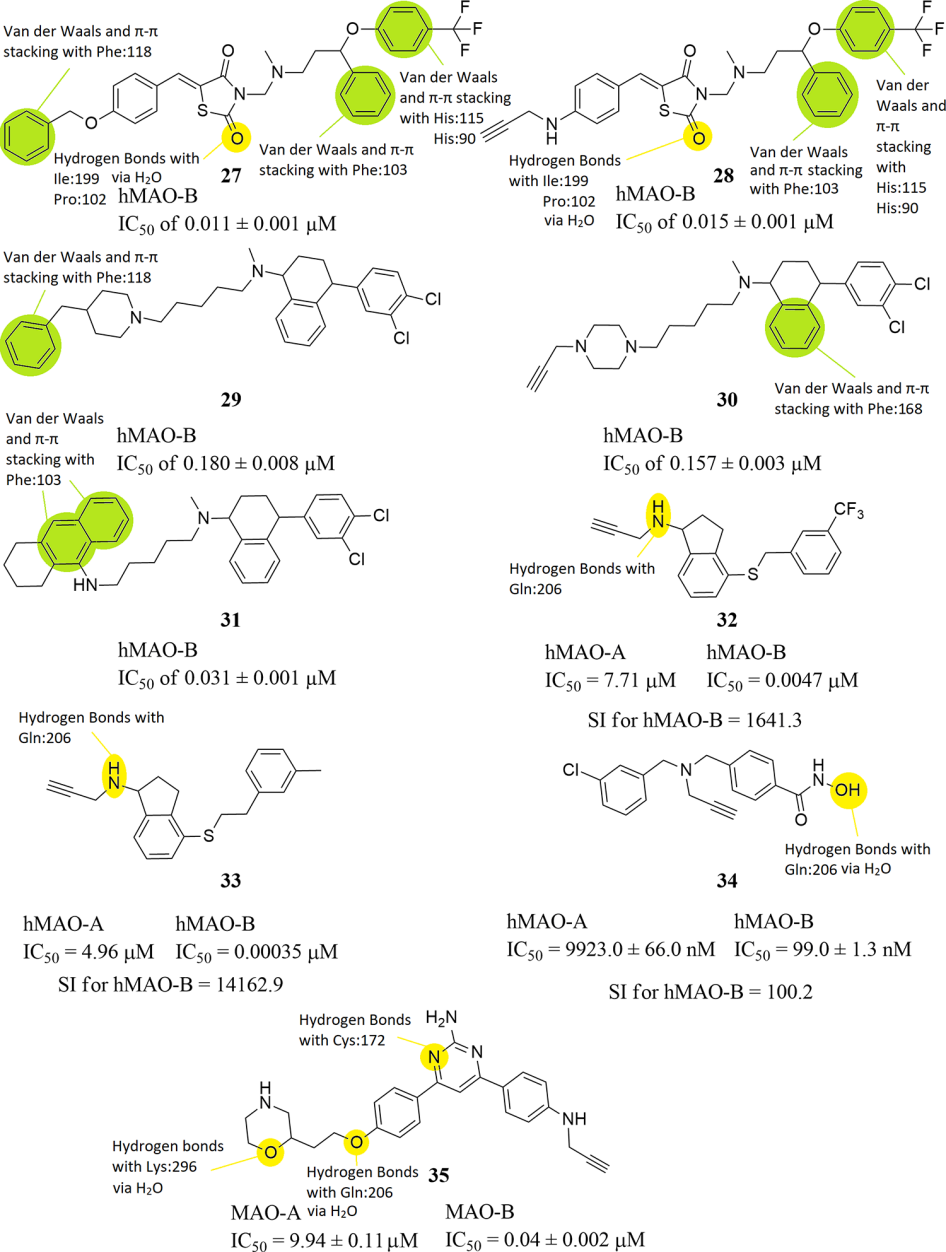
Structures of compounds **27** through **35** and their half maximal inhibitory concentrations (IC_50_). The structure–activity relationships (SARs) of these molecules with hMAO-B are highlighted as well.

1-(Prop-2-yn-1-ylamino)-2,3-dihydro-1*H*-indene-4-thiol derivatives as hMAO-B inhibitors, which were designed by employing a fragment-based drug design strategy to link rasagiline to hydrophobic fragments, were synthesized by Kong et al. as anti-Parkinson’s disease (PD) drugs. Since some of these molecules were excellent selective inhibitors of hMAO-B, they could also be used for the treatment of Alzheimer’s disease. Among the synthesized 31 compounds, compounds **32** (hMAO-A; IC_50_ = 7.71 μM and hMAO-B; IC_50_ = 0.0047 μM with SI for hMAO-B = 1641.3) and **33** (hMAO-A; IC50 = 4.96 μM and hMAO-B; IC_50_ = 0.00035 μM with SI for hMAO-B = 14162.9) (Figure 11) demonstrated very encouraging hMAO-B inhibitory activities and selectivity over hMAO-A, better than rasagiline (hMAO-A; IC50 = 1.01 μM and hMAO-B; IC_50_ = 0.006 μM with SI for hMAO-B = 168.3) and safinamide (hMAO-A; IC_50_ = 223.5 μM and hMAO-B; IC_50_ = 0.098 μM with SI for hMAO-B = 2280.6). *In vitro* studies indicated that **32** and **33** are nontoxic to nervous tissue cells and they have considerable effects against ROS formation and potential neuroprotective activity. All these experimental results suggest that compounds **32** and **33** can be promising candidates for further research for treatment of AD.^82^ From SAR studies it is revealed that the amine- group of compounds **32** and **33** form hydrogen bonds with residue Gln206 (Figure 11). Lipophilic interactions, also, take place between both compounds and the nearby amino acids inside the active site of the enzyme.

### 3.3 2022

Yao et al. synthesized a series of *N*-propargylamine-hydroxamic acid/*o*-aminobenzamide hybrids inhibitors as MTDLs, which also inhibit hMAO-B. These molecules, combining the typical pharmacophores of hydroxamic acid/*o*-aminobenzamide and propargylamine, were designed and synthesized for the treatment of Alzheimer’s disease. Many of the hybrids synthesized, showed moderate to strong hMAO-B inhibitory effects. Among them, hybrid **34** demonstrated the highest potency against hMAO-B (MAO-B, IC_50_ = 99.0 ± 1.3 nM) and exhibited excellent selectivity for hMAO-B (MAO-A, IC_50_ = 9923.0 ± 66.0 nM; SI (Selective Index) for hMAO-B = IC_50_ (hMAO-A)/IC_50_ (hMAO-B) = 100.2). The reference compound used was pargyline (hMAO-B; IC_50_ = 116.8 ± 0.3 nM). In addition, compound **34** (Figure 11) notably reversed Aβ1–42-induced damage in PC12 cells and reduced the production of intracellular ROS, demonstrating strong antioxidant properties. Furthermore, hybrid **34** quickly crossed the blood–brain barrier, accumulated in brain tissue, and significantly improved cognitive dysfunction in the Morris water maze ICR mice model. In conclusion, the inhibitor **34** shows great potential as a therapeutic agent for Alzheimer’s disease.^63^

Kumar et al. reported the synthesis and screening of 24 *N*-propargyl-substituted diphenylpyrimidine derivatives as MTDLs that also inhibit monoamine oxidase enzymes. In this series, **35** (Figure 11) showed the most potent MAO-B inhibitory activity with an IC_50_ value of 0.04 ± 0.002 μM and high selectivity over MAO-A (9.94 ± 0.11 μM) when compared with reference compound pargyline (hMAO-B; IC_50_ = 0.015 ± 0.002 μM). In the reactive oxygen species inhibition studies, **35** lowered intracellular ROS levels in SH-SY5Y cells by 36%. This compound series also demonstrated strong neuroprotective potential, showing up to 90% recovery from 6-hydroxydopamine-induced neuronal damage in SH-SY5Y cells and were found to be irreversible inhibitors and showed no cytotoxicity against neuronal cells. Thus, *N*-propargyl-substituted diphenylpyrimidines displayed drug-like characteristics and have the potential to be developed as MTDLs for the effective treatment of AD.^23^ From SAR studies it is revealed that the hydroxyl- moiety of the hydroxylamine- group of compound **34** forms hydrogen bonds with residue Gln206 via a water molecule, while the same type of bonds are observed between the oxygen of the phenoxy- group of compound **35** with Gln206 via a water molecule (Figure 11). Furthermore, compound **35** develops hydrogen bonds between the oxygen atom of the morpholine- group and Lys296 and the nitrogen atom of the pyrimidine- group and Cys172. Lipophilic interactions, also, take place between both compounds and the nearby amino acids inside the active site of the enzyme.

Chrienova et al. designed, synthesized, and evaluated 24 novel compounds bearing tetrahydroacridine and *N*-propargyl moieties *in vitro*, as MTDLs which also inhibit hMAO-B. Regarding MAO inhibition, compounds **36**, **37**, and **38** (Figure 12) demonstrated the highest inhibitory potential toward hMAO-B (IC_50_ = 162.65 ± 17.35, 40.39 ± 5.98, and 169.95 ± 7.75 nM, respectively). The reference compound used was pargyline (hMAO-B; IC_50_ = 80 ± 1 nM). 7-Phenoxy-*N*-(prop-2-yn-1-yl)-1,2,3,4-tetrahydroacridin-9-amine hydrochloride (**37**) has been identified as a permeable agent that shows a balanced pharmacological profile (inhibits both hMAO-B and other key enzymes for Alzheimer’s disease), becoming a new hit-ligand that deserves further investigation.^64^ From SAR studies it is revealed that van der Waals and π–π stacking interactions between the phenyl- moieties of the quinolone- groups of compounds **36**, **37** and **38** and Tyr326 are developed (Figure 12). Lipophilic interactions, also, take place between these compounds and the nearby amino acids inside the active site of the enzyme.

**Figure 12:**
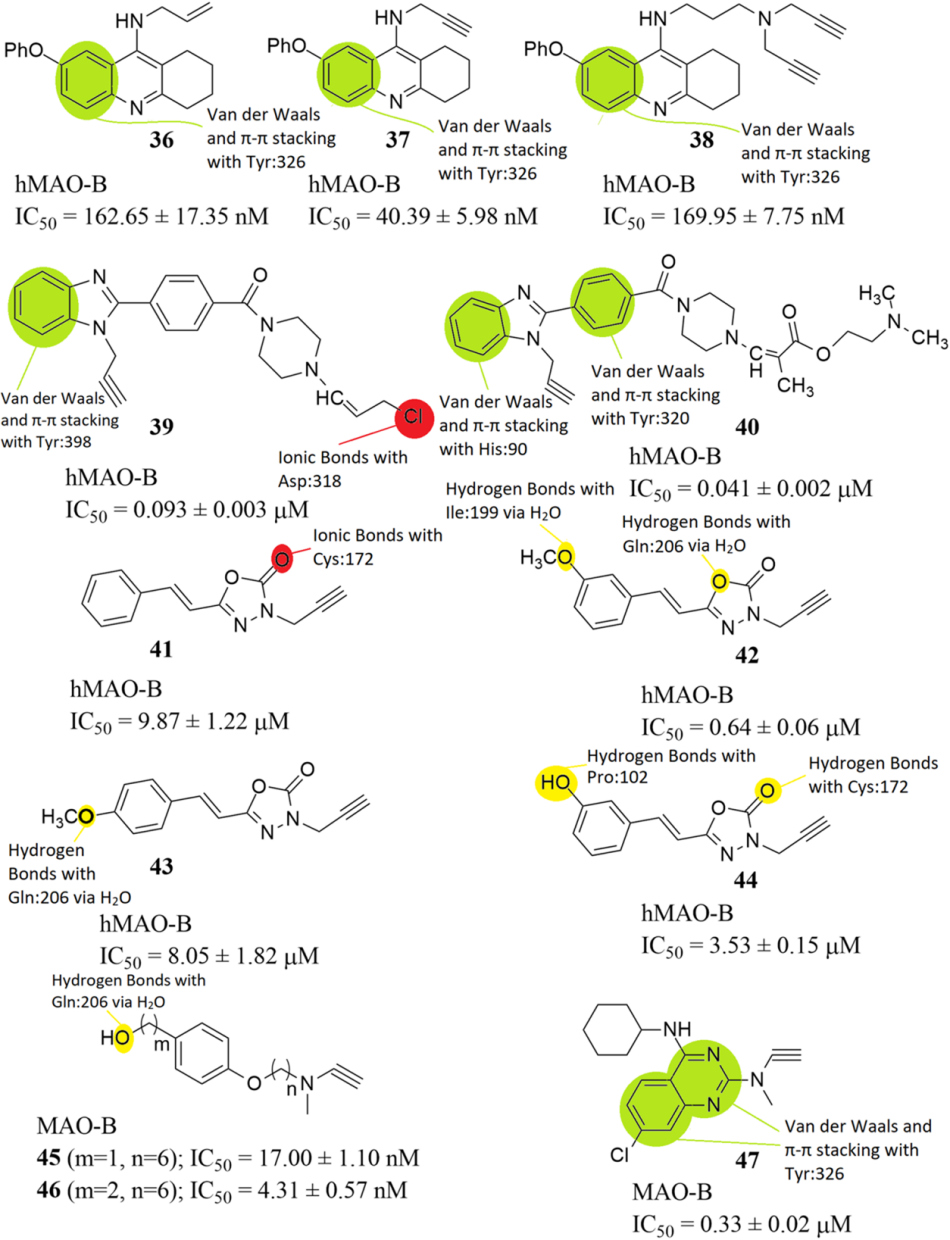
Structures of compounds **36** through **47** and their half maximal inhibitory concentrations (IC_50_). The structure–activity relationships (SARs) of these molecules with hMAO-B are highlighted as well.

Osmaniye et al. designed, synthesized, and evaluated *in vitro* novel benzimidazole derivatives as MTDLs which can also inhibit hMAO-B. Compounds **39** (IC_50_ 0.093 ± 0.003 μM) and **40** (IC_50_ 0.041 ± 0.002 μM) (Figure 12) showed inhibitory activity against MAO-B and compound **40** had inhibition potential similar to that of the reference compound selegiline (IC_50_ 0.037 ± 0.001 μM). Moreover, inhibition tests of Aβ plaque aggregation were performed by means of the *in vitro* kit procedure and compound **40** was found to significantly inhibit this procedure.^83^ From SAR studies it is revealed that van der Waals and π–π stacking interactions between the phenyl- moieties of the 1*H*-benzo­[*d*]­imidazole- groups of compound **39** and Tyr398 and compound **40** and His90 are developed (Figure 12). Compound **40** also develops such interactions between the phenyl- moiety of the acetophenone- group and Tyr320. On the other hand, compound **39** is one of the few compounds in this review that develops ionic bonds, and specifically between its chlorine atom and the amino acid residue Asp318 (Figure 12). Lipophilic interactions, also, take place between both compounds and the nearby amino acids inside the active site of the enzyme.

Herrera-Arozamena et al. synthesized new resveratrol-based multitarget-directed ligands by replacing a phenolic ring of (*E*)-resveratrol with an 1,3,4-oxadiazol-2­(3*H*)-one heterocycle. Compounds **41** (IC_50_ 9.87 ± 1.22 μM), **42** (IC_50_ 0.64 ± 0.06 μM), **43** (IC_50_ 8.05 ± 1.82 μM) and **44** (IC_50_ 3.53 ± 0.15 μM) (Figure 12) showed the best inhibitory activities between the synthesized compounds, for hMAO-B and enzymatic assays on MAO-B showed that they have a reversible behavior, and therefore, they would avoid the side effects of irreversible inhibitors. (*E*)-resveratrol (hMAO-B; IC_50_ = 29.9 ± 1.8 μM) and iproniazid (hMAO-B; IC_50_ = 7.5 ± 0.4 μM) were used as reference compounds. While compound **42** had the lowest IC_50_ value, compound **43** was considered for further evaluation, since it had the most balanced pharmacologic profile. Compound **43** showed the best ability to promote hippocampal neurogenesis, it had a good drug-like profile (positive *in vitro* central nervous system permeability, good physiological solubility, no glutathione conjugation, and lack of PAINS or Lipinski alerts) and exerted neuroprotective and antioxidant actions in both acute and chronic Alzheimer models using hippocampal tissues. Thus, **43** is an interesting MTDL that could stimulate defensive and regenerative pathways and block early events in neurodegenerative cascades.^84^ From SAR studies it is revealed that hydrogen bond interactions between Gln206 and the oxygen atom of the 1,3,4-oxadiazole- group of compound **42** are developed via a water molecule, as well as with the oxygen atom of the phenoxy- group of compound **43** via a water molecule. Compound **42** develops hydrogen bonds with Ile199 via a water molecule, as well. Compound **41** develops ionic bonds via its carbonyl- group with Cys172. Finally, compound **44** establishes hydrogen bonds with Pro102 via its hydroxyl- group and Cys172 via its carbonyl- group. Lipophilic interactions, also, take place between these compounds and the nearby amino acids inside the active site of the enzyme.

### 3.4 2023

To discover novel inhibitors, Zhang et al. designed and synthesized a series of isoform-selective MAO inhibitors with carbon chains of different lengths, and evaluated their enzyme inhibitory activities for MAO-B, cytotoxicity for PC12 cells, abilities against H_2_O_2_-induced cell apoptosis and reactive oxygen species (ROS) production. They also determined their blood–brain barrier permeability by a parallel artificial membrane permeability assay and performed a docking study to verify the relationship between their isoform-selective inhibitory activity and their molecular structure. The compounds that were synthesized combined the *N*-methyl-propargylamine moiety, which can be found in a lot MAO inhibitory drugs (such as selegiline and pargyline), with the natural product salidroside, which possess antioxidation, anti-inflammation, and neuroprotective properties,^85,86^ or tyrosol scaffold, which possess the same properties^87,88^ and exerts a protective effect against dopaminergic neuronal cell death *in vitro*.^89^ Tyramine compounds were also synthesized combining the propargylamine moiety. Among all studied compounds, **45** (MAO-B, IC_50_ = 17.00 ± 1.10 nM) and **46** (MAO-B, IC_50_ = 4.31 ± 0.57 nM) showed an excellent MAO-B affinity when compared with reference compound pargyline (hMAO-B; IC_50_ = 68.72 ± 7.97 nM), thus showcasing the importance of the carbon chain’s length between the alcohol and *N*-methyl-propargylamine moiety in MAOs isoform-selective inhibition (Figure 12). Moreover, **45** had good blood–brain barrier permeability and very low toxicity against PC12 cells, also protecting cells from H_2_O_2_-induced injury, while from flow cytometric experiments these compounds protected PC12 cells from H_2_O_2_-induced apoptosis and intracellular ROS production. Thus, **45** shows promise for the treatment of MAO-B-related disorders, such as Alzheimer’s disease (AD) and Parkinson’s disease (PD).^45^ From SAR studies it is revealed that hydrogen bond interactions between Gln206 and the hydroxyl- group of compounds **45** and **46** via the contribution of a water molecule are developed (Figure 12). Lipophilic interactions, also, take place between both of these compounds and the nearby amino acids inside the active site of the enzyme.

Svobodova et al. designed, synthesized, and biologically evaluated 24 novel *N*-methylpropargylamino-quinazoline multitarget directed ligands, which also inhibit MAO-B. From the results, compound **47** (Figure 12) was the best candidate endowed with a selective MAO-B inhibition (IC_50_ = 0.33 ± 0.02 μM). The reference compound used was pargyline (hMAO-B; IC_50_ = 0.08 ± 0.01 μM). Regarding the mechanism of action, clorgyline and pargyline were tested in order to verify the irreversibility of their action toward the MAO enzymes. As expected, they acted as fully irreversible inhibitors as there was no recovery of enzyme activity. Considering **47**, there was only a partial recovery of the original MAO-B enzyme activity. To confirm the *in silico* predicted ability of **47** to penetrate through the blood brain barrier the parallel artificial membrane-permeability assay (PAMPA) was used, demonstrating a high probability of crossing BBB via passive diffusion. Moreover, the **47** compound lost the cytotoxic effect in the differentiated model of the SH-SY5Y cell line using the MTT assay [MTT: (3-(4,5-dimethylthiazol-2-yl)-2,5-diphenyl-tetraziolium bromide), emphasizing the potential advantage of this compound. On the other hand **47**, revealed significant changes of dehydrogenase activity or glutathione levels when tested at a high concentration (100 μM), while being relatively safe at a lower dose (10 μM). This result is somewhat unexpected, as MAO-B inhibitors typically decrease the production of neurotoxic substances like aldehydes and hydrogen peroxide, which are known to enhance the formation of reactive oxygen species, ultimately leading to greater neuronal damage.^90^ From SAR studies it is revealed that van der Waals and π–π stacking interactions between the quinazoline- group of compound **47** and Tyr326 are developed (Figure 12). Lipophilic interactions, also, take place between the compound and the nearby amino acids inside the active site of the enzyme.

Javed et al. synthesized a framework of hybrids containing pyrimidine/pyrrolidine-sertraline/propargylamine scaffolds as multitarget inhibitors which also inhibit the monoamine oxidase enzymes. From the *in vitro* tested compounds for the MAOs inhibition, compounds **48** and **49** had good IC_50_ values [**48** (hMAO-B; 0.23 ± 0.06 μM) (Figure 13) and **49** (hMAO-B; 0.21 ± 0.06 μM)] and the most potent between them was compound **49**. The standard drug safinamide, used as reference, showed IC_50_ value 0.025 ± 0.003 μM for the hMAO-B inhibitor. After this, *in vivo* experiments in mice were performed. The approximate lethal dose of synthesized compounds **48** and **49** in the experimental mice was 500 mg/kg. Four of the six mice showed stronger evasive behavior and spontaneous activity after the administration/injection at various doses which were ranging from 50 to 2000 mg/kg body weight. The same mice also performed better during escape attempts, and there was an increase in anaphylaxis (measured as aggression during medication and a significant increase in irritation). Five out of the 6 test animals were found to be sleepy at dosages higher than 500 mg/kg. All the animals/mice seemed normal between 24 h and 1 week after the treating with no observable changes in their appearance, activity, or behavior. Compound **48** showed comparatively low permeation from the BBB, while compound **49** showed high BBB permeation. In this study streptozotocin (STZ) was utilized for the creation of a mice model with Alzheimer’s disease and cognitive tests such as the Open Field Test (OFT), the Elevated plus maze Test (EPMT), the Morris Water Maze Test (MWMT) and the Passive Avoidance Test (PAT) were performed after the administration of compounds **48** and **49**. The OFT test for the assessment of locomotor activity revealed a noticeable reduction in the animal’s latency time (interval of time elapsing between a stimulus and a response) at a dose of 80 mg/kg, when compared to the positive control group (administered drugs safinamide or donepezil) and the streptozotocin-treated group. In the EPMT test the measure for the anxiety related behavior of the tested animals was evaluated against piracetam induced learning and memory consolidation. Streptozocin caused a decline in learning and memory consolidation, as evidenced by longer transfer latencies (TLs) on both day 1 (trial) and day 2 (test) compared to the control group. The memory and learning task was successfully retained, as indicated by the transfer latency (TL) on the second day. All mice treated with the synthesized compounds showed improved memory. The MWMT test is highly effective in identifying learning difficulties, spatial memory, and dimensional recall. The reduction in escape latency over several days indicated an improvement in the performance of mice across all groups, except of the streptozotocin group (which was supposed to decline). Regarding the PAT test, with the exception of the Streptozotocin group, the calculations revealed that the retention latency time increased in all the experimental groups showing significant improvement in all animals behavior.^91^ From SAR studies it is revealed that van der Waals and π–π stacking interactions between the phenyl- moieties of the tetralin- groups of compounds **48** and **49** and Tyr398 are developed (Figure 13). Lipophilic interactions, also, take place for both of these compounds and the nearby amino acids inside the active site of the enzyme.

**Figure 13:**
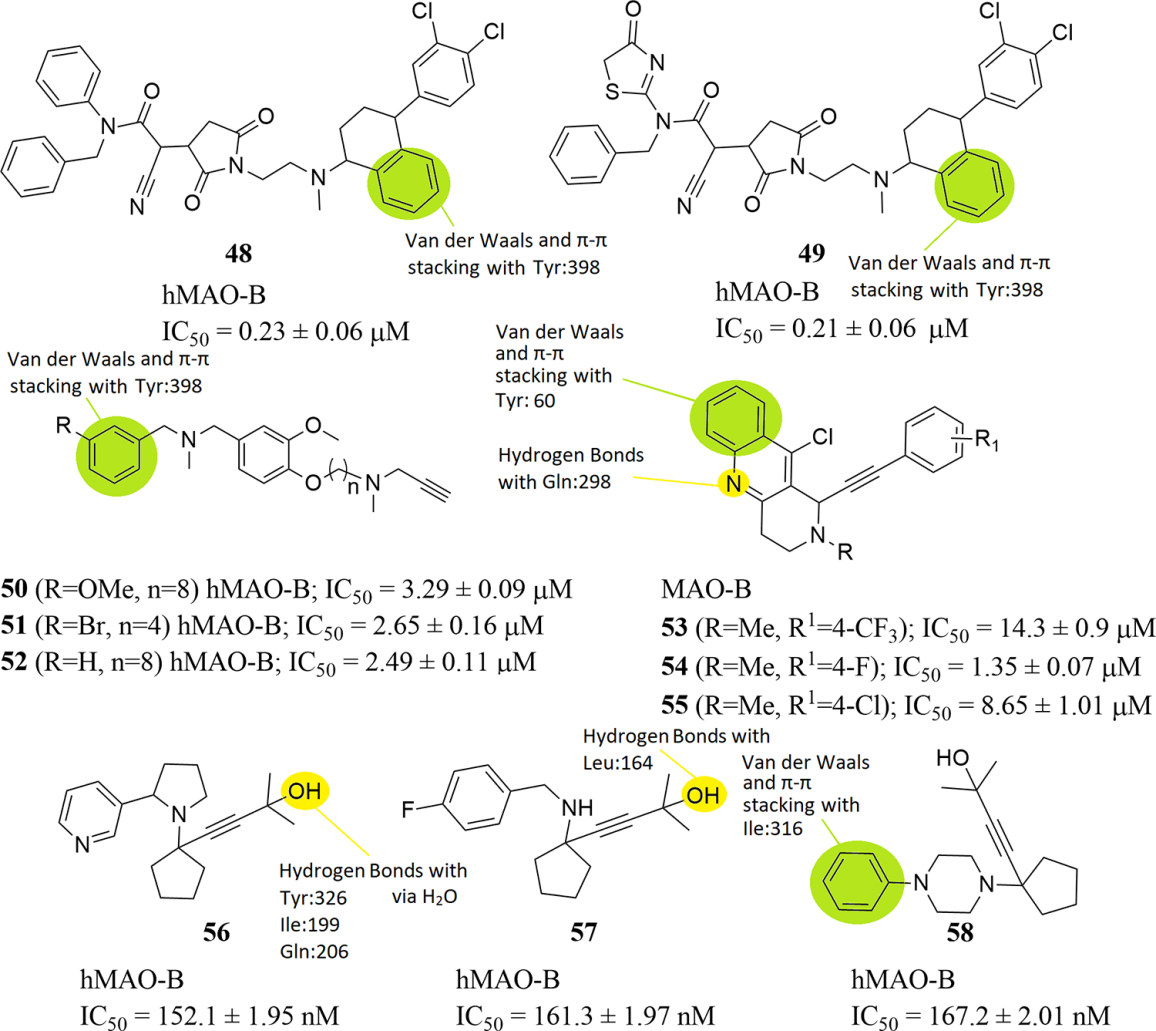
Structures of compounds **48** through **58** and their half maximal inhibitory concentrations (IC_50_). The structure–activity relationships (SARs) of these molecules with hMAO-B are highlighted as well.

Zhong et al. designed, synthesized and investigated *in vitro*, for their inhibition of cholinesterases and monoamine oxidases, novel AP2238-clorgiline hybrids as multitarget agents. Compound **50** showed good inhibitory activity for hMAO-B (IC_50_ = 3.29 ± 0.09 μM), although compounds **51** (IC_50_ = 2.65 ± 0.16 μM) and **52** (IC_50_ = 2.49 ± 0.11 μM) showed better inhibitory activity for hMAO-B (Figure 13). The reference compound used was iproniazide (hMAO-B; IC_50_ = 9.12 ± 0.43 μM). However, because **50** showed the most balanced inhibitory potential against all studied enzymes, it was considered with the best properties. Compound **50** exhibited no toxicity on neural cells, PC12 and BV-2, at 12.5 μM and no acute toxicity at a dosage of 2500 mg/kg. Moreover, **50** can improve the memory function of mice with scopolamine-induced memory impairment and have an excellent ability to cross the blood–brain barrier.^92^ From SAR studies it is revealed that van der Waals and π–π stacking interactions between the phenyl- moieties of the benzylamine- groups of compounds **50**, **51** and **52** and Tyr398 are developed (Figure 13). Lipophilic interactions, also, take place between these compounds and the nearby amino acids inside the active site of the enzyme.

Kulikova et al. synthesized 2-alkyl-10-chloro-1,2,3,4-tetrahydrobenzo­[*b*]­[1,6]­naphthyridines, which possess an internal triple bond in their structure. Compounds **53–55** (Figure 13) proved to be MAO-B inhibitors with potency in the low micromolar range. In particular, the 1-(2-(4-fluorophenyl)­ethynyl) analog **54** achieved an IC_50_ of 1.35 ± 0.07 μM, a value close to that of the well-known MAO-B inhibitor pargyline (2.69 ± 0.48 μM) which was used as reference. This is the first time that a compound with an internal triple bond is found to possess a high inhibitory potency against MAO-B, opening new prospects in the treatment of Alzheimer’s disease via inhibition of the monoamine oxidase enzyme, MAO-B.^93^ From SAR studies it is revealed that residue Gln298 develops hydrogen bonding interactions with the nitrogen atoms of the quinolone- groups of compounds **53**, **54** and **55** (Figure 13). van der Waals and π–π stacking interactions between the phenyl- moieties of the quinoline- groups of compounds **53**, **54** and **55** and Tyr60 are established as well. Lipophilic interactions, also, take place between these compounds and the nearby amino acids inside the active site of the enzyme.

### 3.5 2024

Mavroeidi et al. synthesized quaternary propargylamine derivatives and the inhibitory activity of these molecules was evaluated against hMAO-B enzymes.^94^ The IC_50_ values for all the propargylamines synthesized (range from 152.1 to 164.7 nM) was significantly lower, when compared to the IC_50_ value of pargyline as a MAO-B template (2.25 μM) as described from Ramsay et al.^59^ This suggests that these compounds are more effective inhibitors compared to pargyline. Among the tested propargylamines, **56** (hMAO-B; IC_50_ = 152.1 ± 1.95 nM) and **57** (hMAO-B; IC_50_ = 161.3 ± 1.97 nM) demonstrated the most pronounced efficacy against the MAO-B isoform (Figure 13). This increased potency is likely due to their 2-methylbut-3-yn-2-ol moiety. However, propargylamine **58** (hMAO-B; IC_50_ = 167.2 ± 2.01 nM), which also contains this moiety, has a slightly higher IC_50_ value compared to **56** and **57** (Figure 13). Selegiline (hMAO-B; IC_50_ = 5.82 ± 0.04 nM) was used as reference compound. This difference was attributed to the steric hindrance caused by the bulky groups positioned near the moiety in its structure.^94^ This is the first time that a large number of compounds with a nonterminal triple bond are synthesized and utilized for the inhibition of hMAOs, showcasing so low IC_50_ values. Moreover, the authors of the article highlighted the importance of this innovation, as stated in its title: “Are Terminal Alkynes Necessary for MAO-A/MAO-B Inhibition? A New Scaffold Is Revealed”. From SAR studies it is revealed that residues Tyr326, Ile199 and Gln206 develop hydrogen bonding interactions with the hydroxyl- group of compound **56**. Compound **57** also showcases such interactions via its hydroxyl- group with Leu164. Van der Waals and π–π stacking interactions between the phenyl- group of compound **58** and amino acid residue Ile316 are established as well. Lipophilic interactions take place between these compounds and the nearby amino acids inside the active site of the enzyme.

As a result, numerous synthetic compounds with distinct structures can inhibit hMAO-B to varying extents of selectivity and potency. Categorizing the chemical scaffolds based on their affinity for the hMAO-B isoform is a challenging task. The compounds that showed high inhibitory activity toward hMAO-B were **9**, **15**, **18**, **32**, **33**, **37**, with molecule **33** showcasing the best potency (IC_50_ = 0.00035 μM). Between them, compound **9** had the best selectivity toward hMAO-B, with compounds **32** and **33** also being very selective toward this enzyme.

## 4 Computational Experiments

All of the molecules referenced in this review where docked on the hMAO-B enzyme (6FVZ) and we have calculated their binding energies using the computational software Maestro. For the most potent compound of each figure, based on its IC_50_ value, we performed further computational docking studies using Maestro software, in order to assess the *in silico* inhibitory activity of these molecules to hMAO-B enzyme.

### 4.1 Molecular Docking Illustrations and Interaction Diagrams

For the potent compound **15**, docking studies were performed on hMAO-B (PDB crystal structure: 6FVZ) as can be seen from Figure 14, using Maestro software.^95^ Specifically, compound **15** shows −12.441 kcal/mol binding energy. The reference compound deprenyl showed −8.602 kcal/mol, thus indicating weaker binding. In Figure 14, key interactions are suggested by the software and involve hydrogen bonding between compound **15** and amino acid residues Trp119, Glu483 and Tyr326, as indicated by the arrows on the interaction diagram. Moreover, lipophilic interactions take place between compound **15** and the nearby amino acids inside the hydrophobic pocket of the enzyme’s active site.

**Figure 14:**
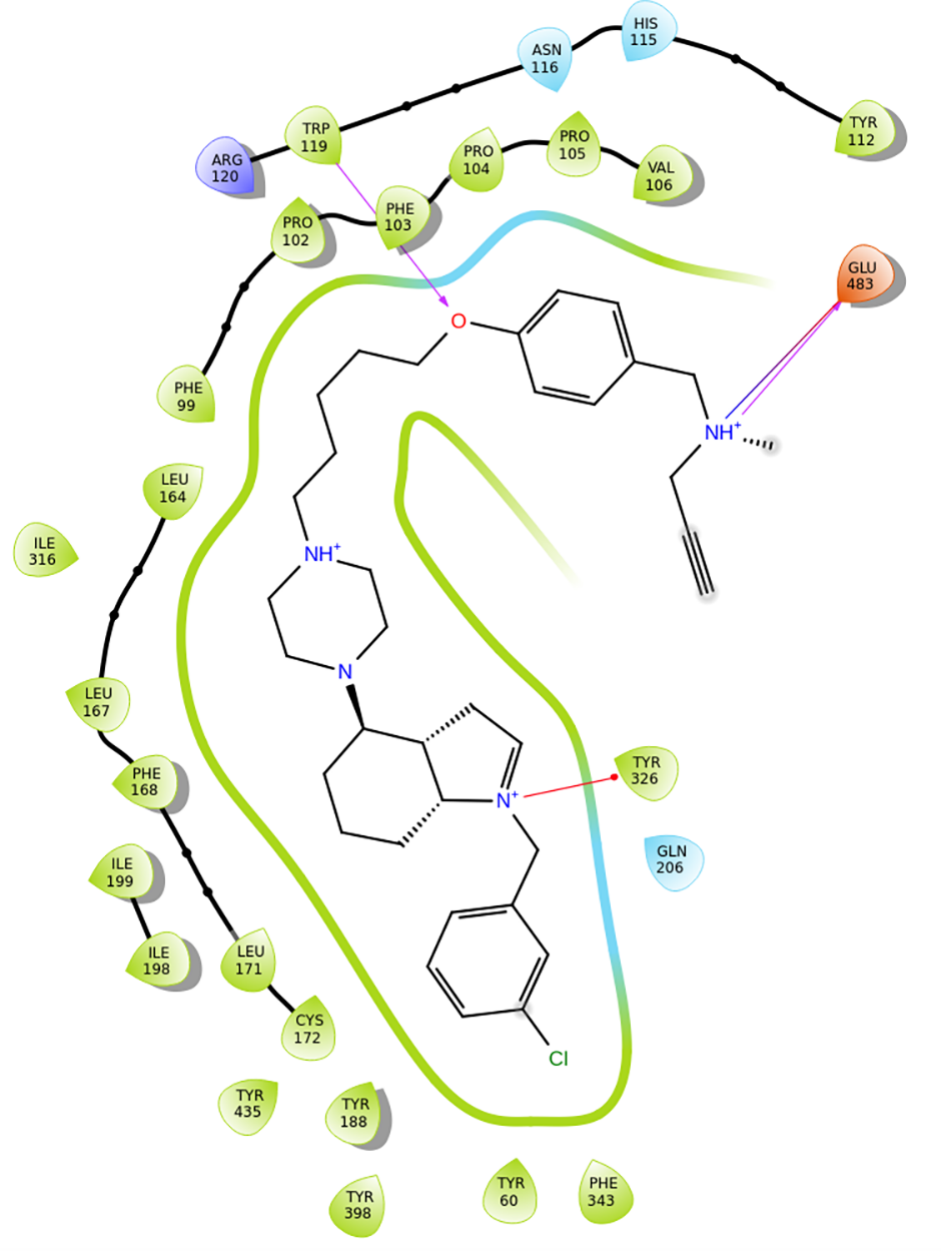
Induced Fit Docking (IFD) with Maestro software of compound **15** and the resulting interactions diagram, where is shown how compound **15** occupies the reactive site of hMAO-B (PDB crystal structure: 6FVZ).

The same procedure was followed for the most potent structure from Figure 10 (compound **18**) as well. Docking studies were performed on hMAO-B (PDB crystal structure: 6FVZ), utilizing this compound, as can be seen from Figure 15, using Maestro software. Specifically compound **18** shows binding energy of −9.526 kcal/mol (the reference compound deprenyl showed −8.602 kcal/mol) and, as a result, it binds strongly to the active site of the enzyme. Hydrogen bonding between Cys172 and the oxygen atom of the naphthoquinone moiety are shown in Figure 15, as indicated by the arrow on the interaction diagram. Moreover, lipophilic interactions can also be observed between compound **18** and the nearby amino acids inside the hydrophobic pocket of the enzyme’s active site.

**Figure 15:**
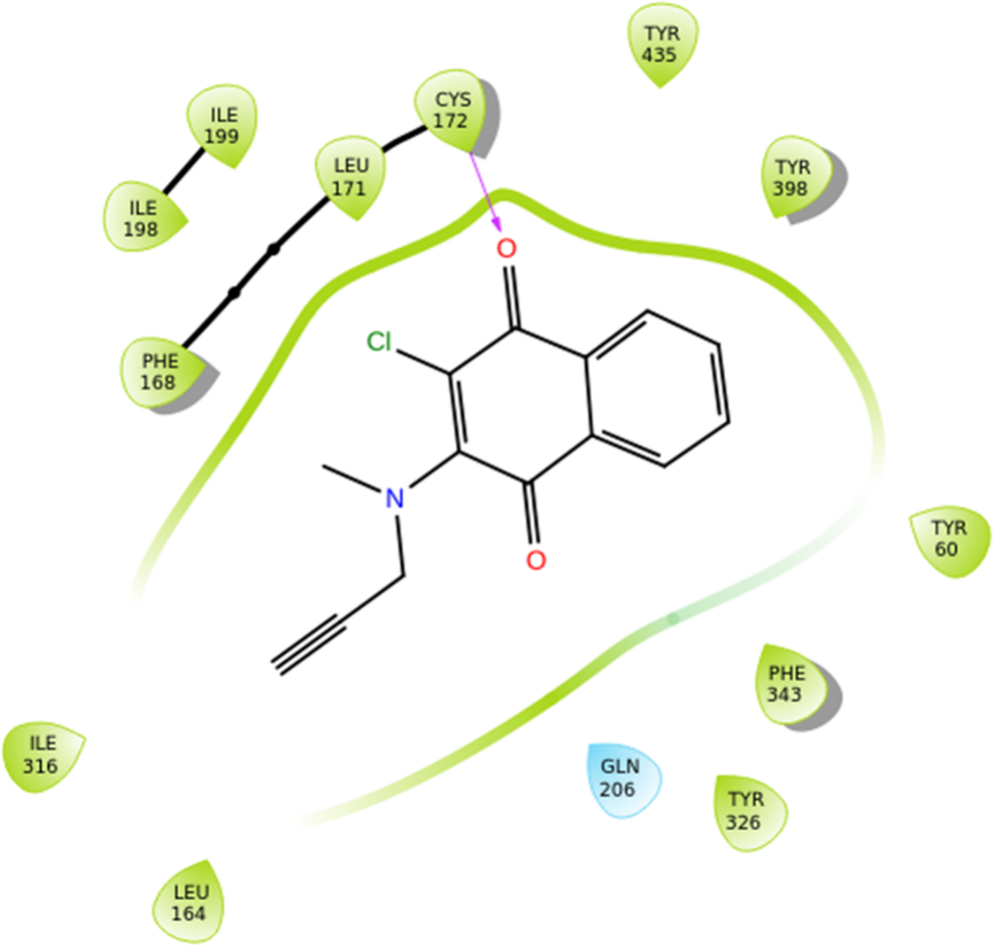
Induced Fit Docking (IFD) with Maestro software of compound **18** and the resulting interactions diagram, where is shown how compound **18** occupies the reactive site of hMAO-B (PDB crystal structure: 6FVZ).

For the most potent structure from Figure 11 (compound **33**), docking studies were performed on hMAO-B (PDB crystal structure: 6FVZ) as can be seen from Figure 16. Specifically, compound **33** shows binding energy −12.876 kcal/mol (the reference compound deprenyl showed −8.602 kcal/mol) and as a result it binds strongly to the active site of the enzyme. In Figure 16, key interactions are suggested by the software and involve hydrogen bonding between compound **33** and amino acid residue Gln206, as indicated by the arrow on the interaction diagram. Moreover, lipophilic interactions take place between compound **33** and the nearby amino acids inside the enzyme’s active site.

**Figure 16:**
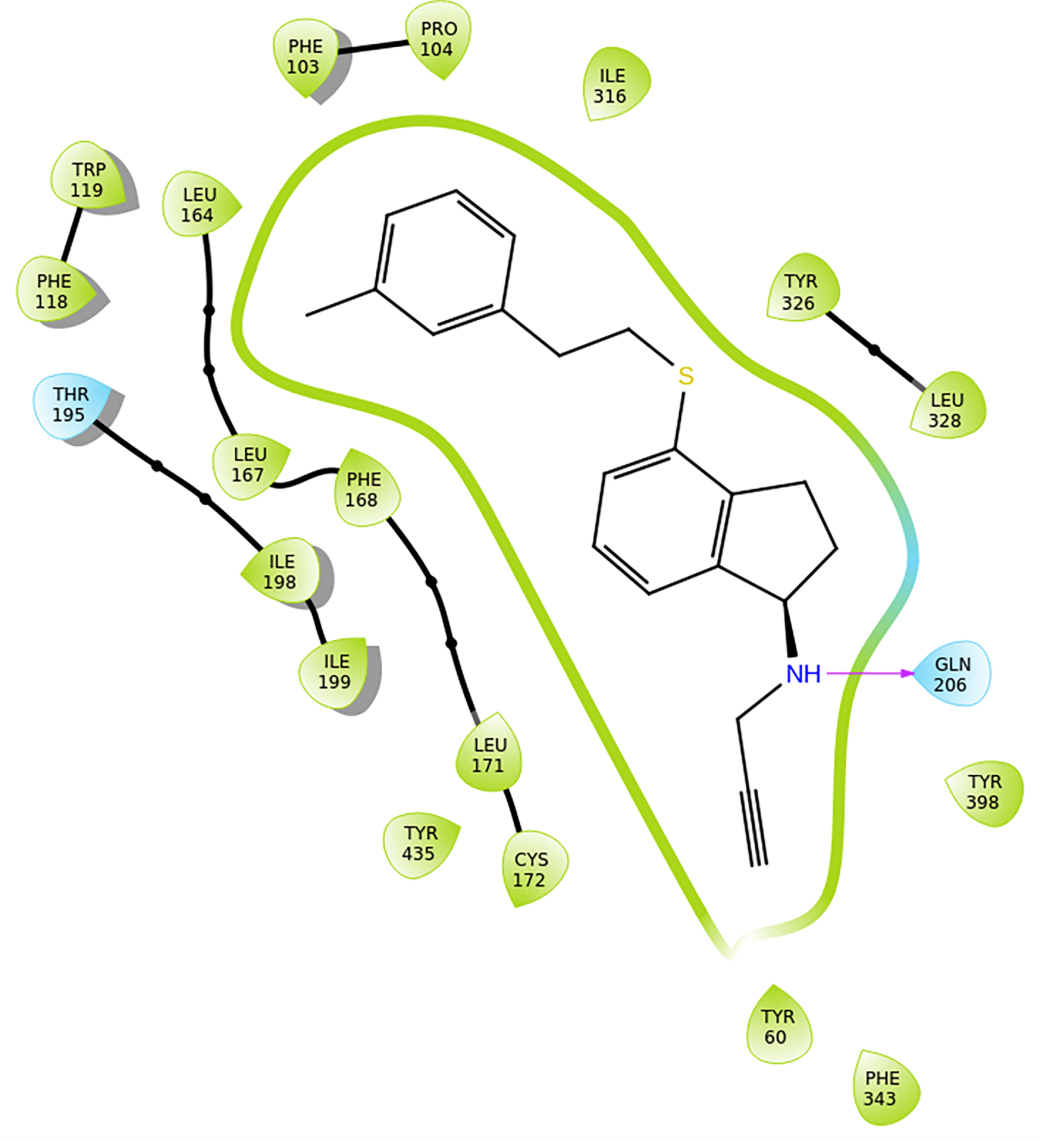
Induced Fit Docking (IFD) with Maestro software of compound **33** and the resulting interactions diagram, where is shown how compound **33** occupies the reactive site of hMAO-B (PDB crystal structure: 6FVZ).

For the most potent structure from Figure 12 (compound **37**), docking studies were performed on hMAO-B (PDB crystal structure: 6FVZ) as can be seen from Figure 17, using Maestro software. Specifically compound **37** shows binding energy −12.511 kcal/mol (the reference compound deprenyl showed −8.602 kcal/mol) and as a result it binds strongly to the active site of the enzyme. Figure 17 highlights several molecular interactions within the protein–ligand complex. Residue Tyr435, is a hydrogen bond donor interacting with the nitrogen atom of the aromatic system, while amino acids Leu171 and Tyr398 are hydrogen bond acceptors interacting with the amine group of the propargylamine moiety. Lipophilic interactions, also, take place between compound **37** and the nearby amino acids inside the active site of the enzyme.

**Figure 17:**
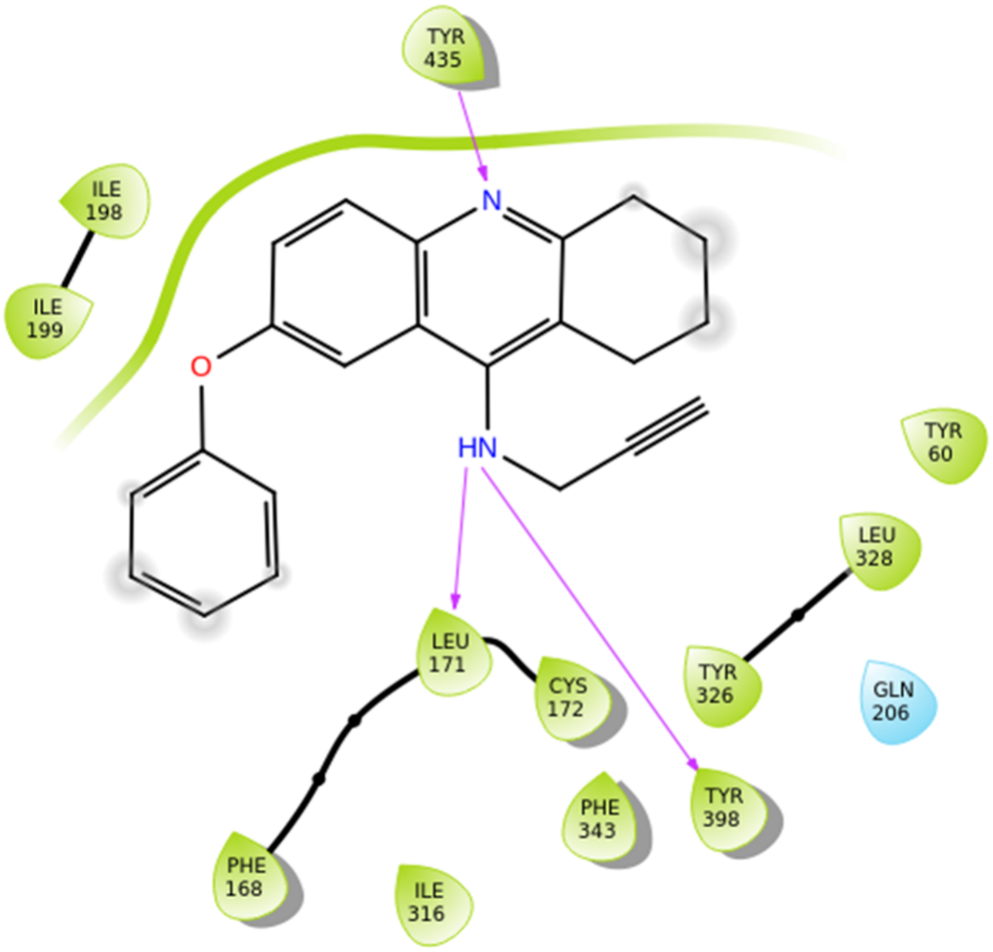
Induced Fit Docking (IFD) with Maestro software of compound **37** and the resulting interactions diagram, where is shown how compound **37** occupies the reactive site of hMAO-B (PDB crystal structure: 6FVZ).

Finally, molecular docking studies were performed for compound **56** (Figure 18), the most potent inhibitor from the new class of inhibitors with a nonterminal propargylamine moiety in its structure. This molecule was docked on hMAO-B (PDB crystal structure: 6FVZ) as can be seen from Figure 18. Specifically compound **56** shows binding energy −10.832 kcal/mol (the reference compound deprenyl showed −8.602 kcal/mol) and as a result it binds strongly to the active site of the enzyme. Figure 18 highlights several molecular interactions within the protein–ligand complex. Residues Gln206, Tyr326 and Ile199, form hydrogen bonds with the hydroxyl- group of compound **56**, via the participation of a water molecule inside the hydrophobic pocket of the enzyme’s active site. Lipophilic interactions, also, take place between compound **56** and the nearby amino acids inside the active site of the enzyme.

**Figure 18:**
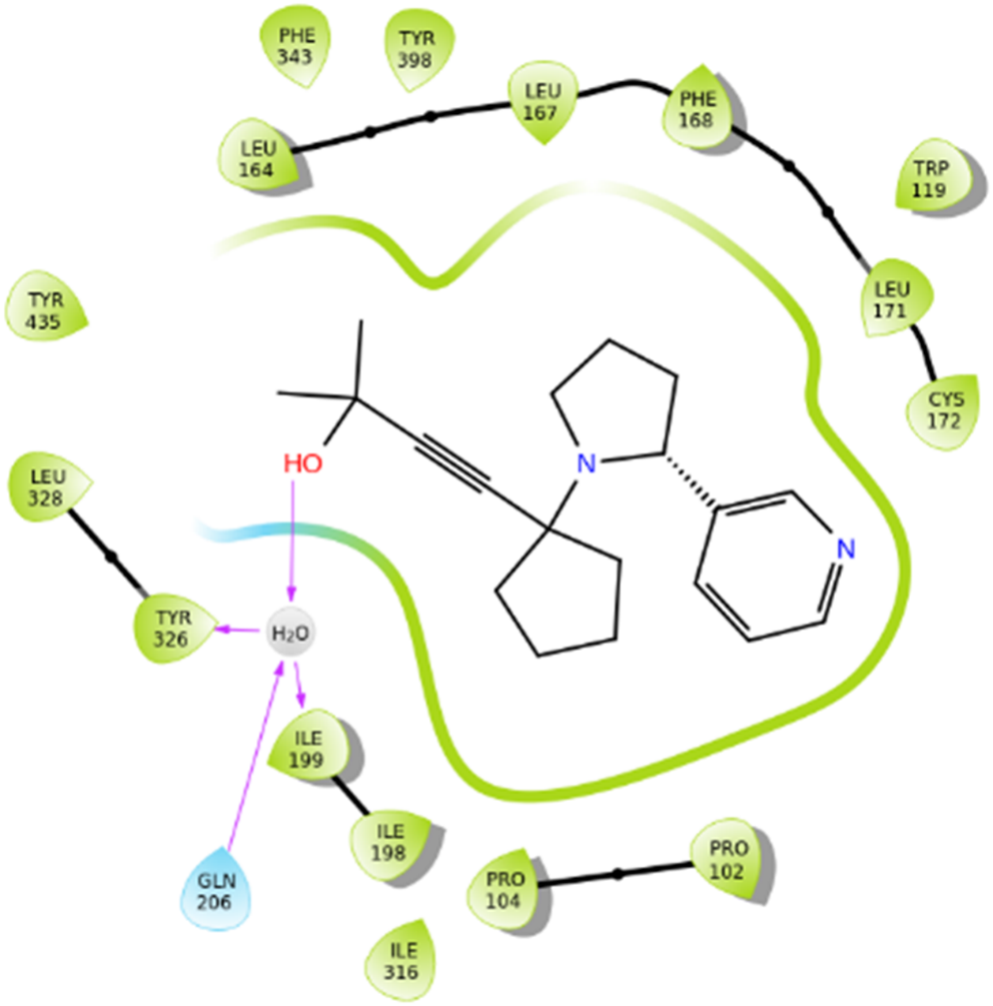
Induced Fit Docking (IFD) with Maestro software of compound **56** and the resulting interactions diagram, where is shown how compound **56** occupies the reactive site of hMAO-B (PDB crystal structure: 6FVZ).

### 4.2 Conformational Relationships

After, the molecules reviewed were docked on the hMAO-B enzyme and classified according to their developed interactions. Class A consists of compounds **1**, **2**, **11**-**13**, **24**, **25**, **32**-**34**, **36**-**38**, **42**, **43**, **45**-**47**, and **56** which suggest interactions with one or more of the amino acids Gln206, Pro102, Ile199 and Tyr326. Class B consists of compounds **14** and **16** which suggest interactions with one or more of the amino acids Gln206, Tyr435, Ile198, Trp119, Phe103, Thr196, and Tyr326. Class C consists of compounds **27** and **28** and suggest interactions with one or more of the amino acids Phe118, Ile198, Pro102, Phe103, His90, and His115. Class D consists of compounds **22** and **23** which suggest interactions with one or more of the amino acids Gln206 and Phe343. Class E consists of compounds **39** and **48**-**52** which show interactions with one or more of the amino acids Tyr398 and Asp118. Class F consists of compounds **26** and **41** which suggest interactions with amino acid Cys172. Finally, Class G consists of the remaining compounds (**17**, **20**, **21**, **26**, **29**-**31**, **35**, **40**, **41**, **44**, **53**-**55**, **57**, and **58**), every one of which suggest interactions with a different kind of residue when compared with the rest. According to the interaction diagrams, the most common kind of interaction of all these molecules, is with amino acid Gln206 which suggests that this amino acid plays a pivotal role in the active site of the enzyme. Another amino acid which seems to play an important role to the overall inhibitory activity of most of these molecules is Tyr326 either through van der Waals interactions and π–π stacking of aromatic rings or via hydrogen bonding. Based on the interactions, it seems that all compounds need to have at least 1 to 3 aromatic rings in the edges of their respective structures in order to develop lipophilic interactions with the hydrophobic pocket of the enzyme’s active site. From comparison of the most potent inhibitors **33** and **37** structures’, it can be observed that in both structures the propargylamine group is on a polycyclic system and that the overall rotational degrees of freedom of these two molecules are less than the others reviewed. When the same kind of analyses is repeated between the strong inhibitors **15** and **18** not a lot of similarities can be observed, except of the fact that the propargylamine moiety of compound **18** is also on a polycyclic system as happens for compounds **33** and **37**. Thus, a thorough conformational analysis needs to be established for these molecules, in order for medicinal chemists to unlock their full potential.

## 5 New Prospects Regarding hMAO-B Inhibition

Based on the studied molecules, the most biologically potent where used for the rational design of compounds **A** and **B** (Figure 19). Compound **A** was designed based on the structures of compounds **32** and **33** (Figure 11), which showcase the lowest IC_50_ values of all terminal propargylamines. Compound **B** was designed based on compounds **56** and **57** (Figure 13) which showcase the lowest IC_50_ values from the nonterminal propargylamine group. Induced fit docking studies were performed for both of these molecules utilizing Maestro software. Specifically compound **A** shows a binding energy of −13.474 and compound **B** shows a binding energy of −10.921 kcal/mol (the reference compound deprenyl showed −8.602 kcal/mol) and as a result they bind strongly to the active site of the enzyme and can serve as drug leads for hMAO-B inhibitors.

**Figure 19:**
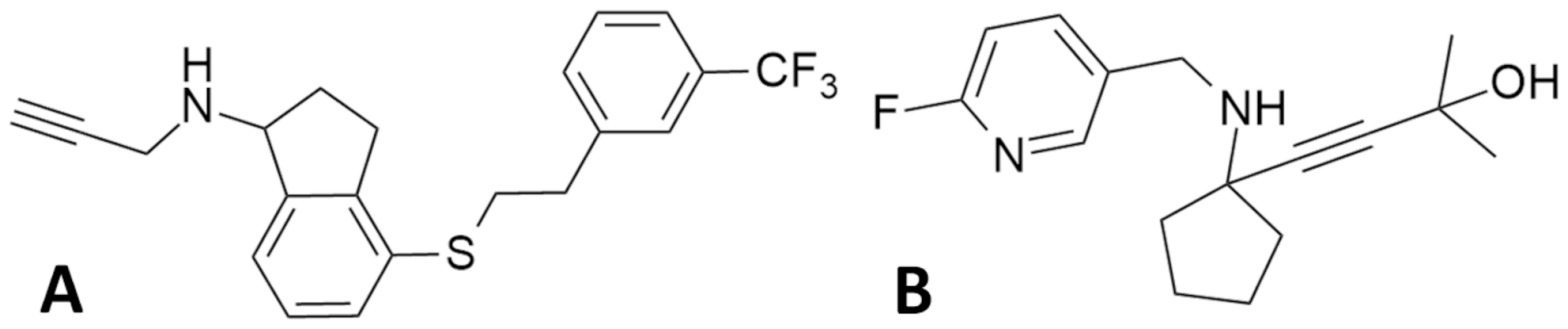
Proposed structures of compounds **A** and **B**.

SwissADME was also utilized in order to compute physicochemical descriptors as well as to predict ADME parameters, pharmacokinetic properties, the druglike nature, and the medicinal chemistry friendliness of these two molecules. Both molecules are in agreement with Lipinski’s rules and their overall physiochemical properties and pharmacokinetics make them good drug candidates and lead molecules for drug discovery (Figure 20).^96^

**Figure 20:**
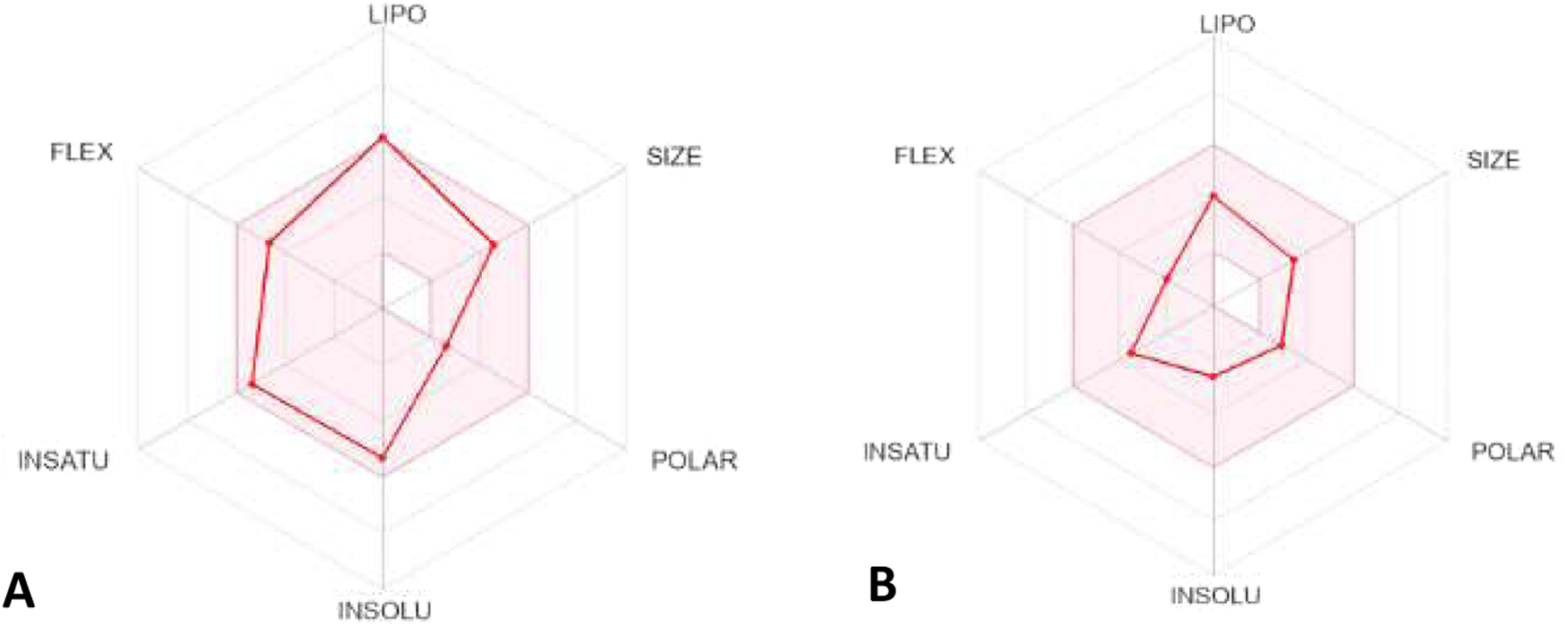
Physicochemical properties of proposed novel compounds **A** and **B**.

Furthermore, some extra compounds which do not possess a propargylamine moiety were tested as hMAO-B inhibitors. Specifically, scopoline and retusin (Figure 21) were found to be constituents of plant *Achillea.* Figure 22 demonstrates the superposition of two ligands, retusin and deprenyl, within the active site of the hMAO-B enzyme. On the left side, the protein structure is shown as a ribbon diagram with helices and loops, highlighting the enzyme’s active site.

**Figure 21:**
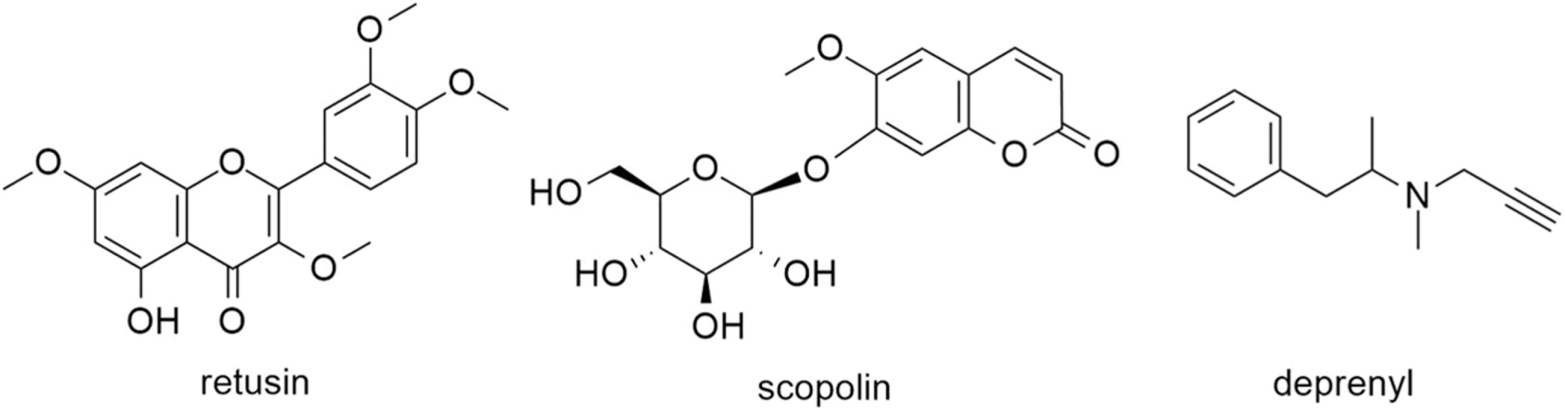
Structures of retusin, scopolin and deprenyl.

**Figure 22:**
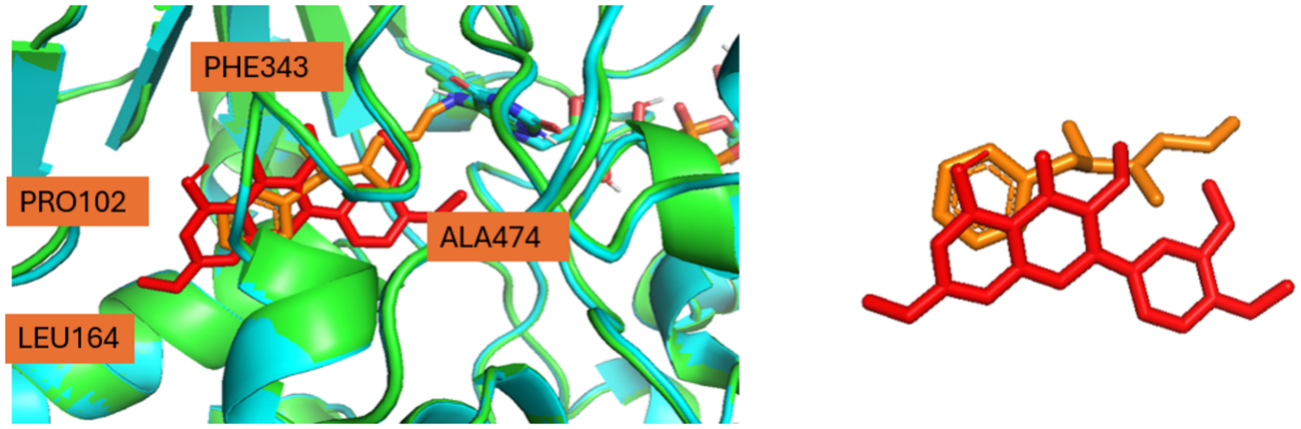
Superposition of retusin with deprenyl inside the active center of hMAO-B (PDB crystal structure: 6FVZ).

The two ligands are overlaid to compare their binding positions and interactions within this site. Retusin is depicted in red, while deprenyl is shown in orange. On the right side, the structure of retusin and deprenyl is presented in a stick representation, illustrating their molecular geometry. This part highlights the ligands’ functional groups and their potential role in interacting with the enzyme. The image is used to study the binding behavior of these new compounds, in comparison to deprenyl, which is an irreversible inhibitor of MAO-B. In Figure 22, the superimposition of retusin (red) and deprenyl (orange) highlights both shared and distinct interactions within the binding pocket of the protein. Common interactions involve residues such as Phe343, which participate in hydrophobic stacking or π–π interactions with both ligands. Similarly, residues like Leu164 contribute hydrophobic stabilization for both compounds.

In Figure 23, the hMAO-B protein is displayed as a ribbon structure with helices and strands showing the enzyme’s overall architecture. Scopolin and deprenyl are superimposed in the active site, with scopolin in blue and deprenyl in orange. In the superimposition of deprenyl (orange) and scopoline (blue), common interactions occur with key residues such as Phe168, Ile198, and Gly205. These residues are central to the binding pocket and likely form hydrophobic or polar interactions with both ligands. Gly292 and Met436 are also shared interaction sites, indicating a conserved binding environment for both compounds. This superposition aids in understanding how different molecules, like scopolin and deprenyl, interact with hMAO-B, revealing critical insights into enzyme inhibition mechanisms and aiding drug design efforts for hMAO-B-related diseases such Alzheimer’s disease.

**Figure 23:**
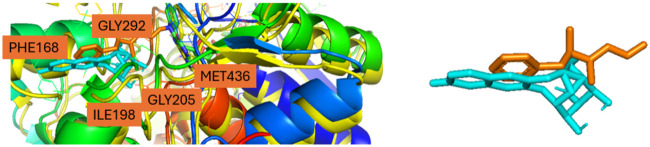
Superposition of scopolin with deprenyl inside the active center of hMAO-B (PDB crystal structure: 6FVZ).

The above examples illustrate the necessity of more research efforts to develop selective hMAO-B inhibitors. As shown from the superimpositions and the new structures docked in the active site there is still a lot of space for development of new leads. Computational chemistry can aid to achieve this aim, as is illustrated from the above examples.

## 6 Conclusions and Future Perspectives

Neuropsychiatric disorders, particularly Alzheimer’s disease, impose a significant psychological and economic burden on both patients and society globally. Age-related AD is triggered by multiple factors. The MAO enzyme catalyzes the breakdown of various neurotransmitter amines, producing harmful neurotoxic byproducts such as H_2_O_2_, NH_3_, and aldehydes, which accelerate the progression of AD. Additionally, activated MAO contributes to the formation of neurofibrillary tangles and the degeneration of cholinergic neurons. MAO has been recognized as a crucial drug target for Alzheimer’s disease treatment, and its inhibitors have significantly enhanced our understanding of aminergic neurotransmission. Evidence showed that MAO plays an important role in brain functionality and development and its inhibitors are effective therapeutic agents of Alzheimer’s disease. In this review, a bibliographic search was conducted for 59 molecules synthesized between 2020 and 2024 that contained the propargylamine moiety in their structure. The compounds reviewed were chosen based on their biological activity and those with the lowest IC_50_ values from each article were selected for discussion. Between them, compound **33** was the most potent inhibitor of hMAO-B, while compound **9** was the most selective one. Based on conformational relationships’ analysis the most common kinds of interactions of all these molecules, are with amino acid Gln206 via hydrogen bonding and Tyr326 either through van der Waals interactions and π–π stacking of aromatic rings or via hydrogen bonding. Moreover, it seems that all compounds need to have at least 1 to 3 aromatic rings in the edges of their respective structures in order to develop lipophilic interactions with the hydrophobic pocket of the enzyme’s active site and exert their potency. Also, from comparison of the most potent inhibitors **33** and **37**, minimization of the overall rotational degrees of freedom near the propargylamine moiety is desirable. Based on the structures of compounds **32** and **33**, potent possible inhibitors of hMAO-B, compounds **A** and **B** were rationally designed. Compound **A** showcased a binding energy of −13.474 kcal/mol (the reference compound deprenyl showed −8.602 kcal/mol) based on docking studies using Maestro software, while compound **B** shows a binding energy of −10.921 kcal/mol. As a result it is suggested that both **A** and **B** compounds bind strongly to the active site of the hMAO-B enzyme and could be used as lead compounds in the future. Furthermore, compounds retusin and scopoline which do not possess a propargylamine moiety were tested (Figures 22 and 23). These molecules are constituents of plant *Achillea* and can serve as lead hMAO-B inhibitors. Based on superimposition studies with the known inhibitor deprenyl, both molecules showed shared and distinct interactions within the binding pocket of the protein. These examples highlight the need for further research to create selective hMAO-B inhibitors.
